# So Closely Related and Yet So Different: Strong Contrasts Between the Evolutionary Histories of Species of the *Cardamine pratensis* Polyploid Complex in Central Europe

**DOI:** 10.3389/fpls.2020.588856

**Published:** 2020-12-18

**Authors:** Andrea Melichárková, Marek Šlenker, Judita Zozomová-Lihová, Katarína Skokanová, Barbora Šingliarová, Tatiana Kačmárová, Michaela Caboňová, Matúš Kempa, Gabriela Šrámková, Terezie Mandáková, Martin A. Lysák, Marek Svitok, Lenka Mártonfiová, Karol Marhold

**Affiliations:** ^1^Institute of Botany, Plant Science and Biodiversity Centre, Slovak Academy of Sciences, Bratislava, Slovakia; ^2^Department of Botany, Faculty of Science, Charles University, Prague, Czechia; ^3^Central European Institute of Technology (CEITEC), Masaryk University, Brno, Czechia; ^4^Department of Experimental Biology, Faculty of Science, Masaryk University, Brno, Czechia; ^5^National Centre for Biomolecular Research (NCBR), Faculty of Science, Masaryk University, Brno, Czechia; ^6^Department of Biology and General Ecology, Faculty of Ecology and Environmental Sciences, Technical University in Zvolen, Zvolen, Slovakia; ^7^Department of Ecosystem Biology, Faculty of Science, University of South Bohemia, České Budějovice, Czechia; ^8^Botanical Garden, Pavol Jozef Šafárik University, Košice, Slovakia

**Keywords:** Brassicaceae, environmental niche, genome size, hybridization, microsatellites, phylogeography, polyploidy, target enrichment

## Abstract

Recurrent polyploid formation and weak reproductive barriers between independent polyploid lineages generate intricate species complexes with high diversity and reticulate evolutionary history. Uncovering the evolutionary processes that formed their present-day cytotypic and genetic structure is a challenging task. We studied the species complex of *Cardamine pratensis*, composed of diploid endemics in the European Mediterranean and diploid-polyploid lineages more widely distributed across Europe, focusing on the poorly understood variation in Central Europe. To elucidate the evolution of Central European populations we analyzed ploidy level and genome size variation, genetic patterns inferred from microsatellite markers and target enrichment of low-copy nuclear genes (Hyb-Seq), and environmental niche differentiation. We observed almost continuous variation in chromosome numbers and genome size in *C. pratensis* s.str., which is caused by the co-occurrence of euploid and dysploid cytotypes, along with aneuploids, and is likely accompanied by inter-cytotype mating. We inferred that the polyploid cytotypes of *C. pratensis* s.str. are both of single and multiple, spatially and temporally recurrent origins. The tetraploid *Cardamine majovskyi* evolved at least twice in different regions by autopolyploidy from diploid *Cardamine matthioli*. The extensive genome size and genetic variation of *Cardamine rivularis* reflects differentiation induced by the geographic isolation of disjunct populations, establishment of triploids of different origins, and hybridization with sympatric *C*. *matthioli*. Geographically structured genetic lineages identified in the species under study, which are also ecologically divergent, are interpreted as descendants from different source populations in multiple glacial refugia. The postglacial range expansion was accompanied by substantial genetic admixture between the lineages of *C*. *pratensis* s.str., which is reflected by diffuse borders in their contact zones. In conclusion, we identified an interplay of diverse processes that have driven the evolution of the species studied, including allopatric and ecological divergence, hybridization, multiple polyploid origins, and genetic reshuffling caused by Pleistocene climate-induced range dynamics.

## Introduction

Polyploidy is a widespread evolutionary phenomenon and a major mechanism of sympatric speciation in plants (Soltis et al., 2003; Coyne and Orr, 2004; Soltis and Soltis, 2009; Soltis et al., 2014). Diploids and their polyploid progeny often coexist at least in initial phases, although later some geographical or ecological shifts may evolve (Soltis and Soltis, 2009; Parisod et al., 2010; Soltis et al., 2014; see e.g., Hülber et al., 2015; Arrigo et al., 2016). Although reproductive isolation is assumed between diploids and related polyploids, allowing for independent evolution and speciation of polyploid lineages even in sympatry, it may be incomplete and permits some inter-ploidy gene flow (Sonnleitner et al., 2013; Kolář et al., 2017; Sutherland and Galloway, 2017; Baduel et al., 2018). Recurrent polyploid formation and weak reproductive barriers between cytotypes or polyploid lineages generate intricate polyploid species complexes with reticulate evolutionary histories (e.g., Bardy et al., 2010; Ma et al., 2010; Frajman et al., 2016; Španiel et al., 2017; Mandák et al., 2018; Padilla-García et al., 2018; Melichárková et al., 2019). They frequently show weak genetic separation among polyploid species, discrepancies between morphological and genetic patterns, and shallow, largely unresolved phylogenetic structuring. Several studies of polyploid species complexes in Europe indicate their recent diversification, which has been dated to the Pliocene and, especially, the Pleistocene, driven by repeated cycles of glaciation-induced range shifts, and population isolation in refugia followed by range expansion and secondary contact (Franzke and Hurka, 2000; Bardy et al., 2010; Pachschwöll et al., 2015; Frajman et al., 2016; Dauphin et al., 2018; Melichárková et al., 2019; Rojas-Andrés et al., 2020). High species and genetic diversity has repeatedly been observed in Southern Europe, reflecting allopatric long-term survival in stable glacial refugia and only small-scale range shifts (Nieto Feliner, 2014), whereas a highly dynamic glacial and postglacial history can be expected in Central Europe, shaped by colonization of different lineages from southern refugia, their admixture in contact zones, as well as population survival and expansion from cryptic northern refugia (Hewitt, 2001; Birks and Willis, 2008; Stewart et al., 2010). Therefore, understanding the evolutionary processes that generated the present-day variation patterns, cytotypic and genetic structure in European polyploid species complexes is a challenging task.

To uncover evolutionary processes underlying high diversity inpolyploid complexes, we focus on the *Cardamine pratensis* species complex, which is widespread in Europe and exhibits extensive but poorly resolved variation in Central Europe. It is one of the most complicated polyploid complexes of the family Brassicaceae, comprising sexually reproducing, allogamous perennials capable of vegetative propagation (Lövkvist, 1956) but with no reports of apomixis (which is extremely rare in this family, Brukhin et al., 2019). It includes a number of species and genetic lineages from the diploid to the dodecaploid level, with both aneuploids and dysploids (arisen via chromosomal rearrangements, Mandáková et al., 2013) documented, which makes it an excellent model system for addressing the reticulate evolutionary histories of polyploid complexes (Franzke and Hurka, 2000; Lihová and Marhold, 2006; Marhold et al., 2018 and references therein). Several well-defined and genetically distinct diploid endemic species occur in Southern Europe (Franzke and Hurka, 2000; Lihová et al., 2003; Lihová et al., 2004). Conversely, Central and Northern Europe harbor a series of less differentiated diploid and polyploid populations and species with unresolved relationships, putatively of postglacial origin (see Franzke and Hurka, 2000). Their taxonomic treatment is based on detailed morphological, chromosomal, and ecological analyses, as well as on hybridization experiments (Lövkvist, 1956; Urbanska-Worytkiewicz and Landolt, 1974; Marhold and Záborský, 1986; Marhold, 1994a, b; reviewed by Lihová and Marhold, 2006; Marhold et al., 2018; see also Table 1). In Central Europe, they include diploid *Cardamine matthioli*, tetraploid *Cardamine majovskyi*, highly polyploid *Cardamine dentata*, and the most complicated species *Cardamine pratensis* s.str., that includes diploid to heptaploid populations growing across most of Europe from lowlands to the alpine belt. Several attempts have been made to split this polymorphic species into more entities. Some authors recognized *Cardamine nemorosa* and *Cardamine udicola* in lowland to montane areas (Urbanska-Worytkiewicz and Landolt, 1974), and diploid populations from the foothills of the Eastern Carpathians were informally treated as a morphotype ‘ucranica’ (Marhold, 1994b, 1995). In addition, high-elevation Alpine and Eastern Carpathian populations were attributed to *C. rivularis* by Lövkvist (1956) and Urbanska-Worytkiewicz and Landolt (1974; see Table 1). *Cardamine rivularis*, however, is a morphologically and genetically different species occurring only in the Southern Carpathians (Romania) and in Bulgarian mountains (Marhold, 1995; Franzke and Hurka, 2000). Thus, the erroneously classified high-elevation Alpine and Eastern Carpathian populations later became informally referred to as *C. rivularis* auct. non Schur (e.g., Zozomová-Lihová et al., 2014a). Despite the observed ploidy level variation, morphological, and ecological diversity of these species, all genetic markers applied so far (i.e., allozymes, sequences of nrDNA, cpDNA, RAPDs, and AFLP markers; Franzke and Hurka, 2000; Lihová et al., 2003, 2004) have yielded only low resolution and failed to resolve relationships among Central European populations of the *C. pratensis* complex and within *C. pratensis* s.str. in particular.

**TABLE 1:** Overview of present and previous taxonomic treatments of the *Cardamine pratensis* species complex in Central Europe.

Marhold et al. (2018) and present study	Ploidy level (Kučera et al., 2005)	Distribution range	Lövkvist, 1956	Urbanska-Worytkiewicz and Landolt, 1974	Landolt, 1984	Marhold, 1994a, b	Franzke and Hurka, 2000
*C. matthioli* Moretti	2*x*	Central and SE Europe	*C. matthioli*	*C. matthioli*	*C. matthioli*	*C. matthioli*	*C. matthioli*
*C. majovskyi* Marhold and Záborský	4*x*	Central and SE Europe	–	–	*–*	*C. majovskyi*	*C. majovskyi*
*C. pratensis* L.	2*x*–7*x*	Europe, N Africa, N Asia, introduced to N America	*C. pratensis*	*C. pratensis*	*C. pratensis*	*C. pratensis*	*C. pratensis*
*C. pratensis*	*2x*	Germany, Switzerland, France (lower altitudes)	*C. pratensis*	*C. nemorosa* Lej.	*C. pratensis*	–	*C. pratensis*
*C. pratensis*	*2x*, *4x*	Germany, Switzerland (lower altitudes)	*C. pratensis*	*C. udicola* Jord.	*C. udicola*	*C. pratensis*	*C. udicola*
*C. pratensis*	*2x*	E Carpathians (lower altitudes)	*C. pratensis*	*–*	*–*	*C*. *pratensis* “ucranica” type	–
*C. pratensis*	*2x*, *4x*	Carpathians and Alps [(sub-)alpine belt]	*C. rivularis* Schur	*C. rivularis* Schur	*C. rivularis* Schur	*C*. *pratensis* “rivularis auct.” type	*C. rivularis* auct. non Schur
*C. rivularis* Schur *	*2x*, *3x*	S Carpathians and Bulgaria [(sub-)alpine belt]	–	–	*–*	*C. rivularis*	*C. rivularis*
*C. dentata* Schult. **	(7*x*)8*x*−12*x*	Central and NW Europe	*C. palustris* Petermann	–	*C. palustris*	*C. dentata*	*C. dentata*

*A species growing only in Southeastern Europe, but previously confused with (sub-)alpine populations from the Eastern Carpathians and Alps; sampled and analyzed in the present study. **A species not included in the present study due to its high polyploidy.

**FIGURE 1:**
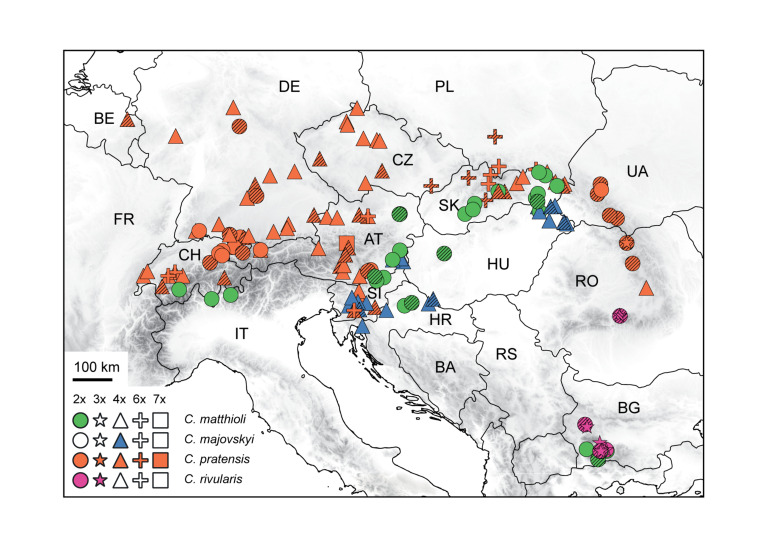
Distribution and ploidy levels of populations of *Cardamine matthioli*, *C. majovskyi*, *C. pratensis*, and *C. rivularis* sampled in the present study. All population samples were used in flow cytometric and microsatellite analyses, while a selection was used in Hyb-Seq analyses (symbols highlighted by hatching). For details on the localities, see Supplementary Data 1.

The aim of this paper is to disentangle the evolution of the *C. pratensis* complex in Central Europe (Figure 1), where much variation among the populations is observed, but with so far unresolved structure and relationships. We analyzed chromosome number, ploidy level and genome size variation, explored genetic patterns based on microsatellite markers and high-throughput target enrichment of low-copy nuclear genes (Hyb-Seq), and reconstructed species’ environmental niches. Microsatellites are efficient, high-resolution genetic markers for both population- and species-level studies (Hodel et al., 2016; Li et al., 2017), and have also been used with success when addressing hybridization events and polyploid evolution (e.g., Sampson and Byrne, 2012; Zozomová-Lihová et al., 2014a, 2015; Feulner et al., 2019; Hu et al., 2019). The Hyb-Seq approach, which captures target exons with flanking intronic and intergenic regions (Weitemier et al., 2014), allows to resolve relationships at various evolutionary levels from the population level up (Villaverde et al., 2018; Tomasello et al., 2020), including polyploids (e.g., Carter et al., 2019). Our specific aims were to (1) resolve the cytotypic and genetic structure of the species under study, (2) identify intraspecific genetic lineages, potentially reflecting descendants from distinct glacial refugia and postglacial colonization routes, (3) explore if and to what extent the lineages correlate with the cytotypic variation and display different environmental niches, (4) resolve single or polytopic polyploid origins, (5) explore the patterns and extent of hybridization between species and lineages, and, finally, (6) to determine how the structure resolved corresponds with previous taxonomic treatments.

## Materials and Methods

### Plant Material

The *C. pratensis* complex occupies wet meadows and pastures, stream banks, springs, and floodplain forests from lowlands up to the alpine belt. We sampled all species recognized in Central Europe (except for *C. dentata*, which was beyond the scope of the present study due to its high polyploidy), and representatively covered their geographic, elevational and cytotypic variation (Figure 1 and Supplementary Data 1). They were identified based on plant morphology and ecology of the sites (mainly elevation), following the concepts of previous authors (see Introduction), with the aid of ploidy level estimation if necessary. In addition, *C. rivularis* growing in Southeastern Europe was also sampled and analyzed here for proper interpretation of the high-elevation Central European populations previously attributed to this species (Table 1), as well as due to its hybridization with *C. matthioli* reported by Ančev et al. (2013, see below).

*Cardamine matthioli* is a diploid (2*n* = 16) distributed in Central Europe and extending to Southeastern Europe (Marhold, 1994b, 2000). Aneuploids with supernumerary chromosomes ranging from 17 to 22 have been observed sporadically (Kučera et al., 2005). The species grows mainly in lowlands and up to the montane belt, and only exceptionally reaches elevations above 1,000–1,500 m (Marhold, 1994b; Ančev, 2006). In total, 223 plants from 28 populations were collected.

*Cardamine majovskyi* is a tetraploid (2*n* = 32) so far reported from two disjunct areas in Central Europe, growing in lowlands and the montane belt up to 600 m above sea level (Marhold et al., 2018 and references therein). Its autotetraploid origin from *C. matthioli* was inferred (Franzke and Hurka, 2000; Lihová and Marhold, 2006; Marhold et al., 2018). Aneuploids with 2*n* = 34 have been rarely observed (Marhold, 1994a). In total, 208 plants from 21 populations were collected.

*Cardamine pratensis* s.str. (further referred to without ‘s.str.’) is widely distributed throughout most of Europe, except its southernmost areas (Marhold, 1994b; Lihová and Marhold, 2006). It grows across a large elevational span, from lowlands up to the alpine belt in the Alps, Carpathians, and the central Pyrenees. It comprises a number of cytotypes ranging from diploid to heptaploid, including also aneuploids and dysploids (Kučera et al., 2005; Mandáková et al., 2013). We sampled 959 plants from 87 populations originating in Central Europe and adjacent areas. These also included populations previously attributed to *C. nemorosa*, *C. udicola*, *C. rivularis* auct., and the ‘ucranica’ morphotype. In the case of these putative taxa, we specifically sampled the same populations or in regions referred to by previous authors (see Supplementary Data 1 for details).

*Cardamine rivularis* occurs in the Southern Carpathians in Romania and in high mountain ranges in Bulgaria, from the montane to the alpine belt (Marhold, 1994b, 1995, 1996; Ančev et al., 2013). The diploid cytotype has so far been recorded in Bulgaria, and both diploids and less frequently also triploids were found in Romania (Marhold, 1994a; Kučera et al., 2005; Ančev et al., 2013). In addition, a triploid hybrid of *C. matthioli* and *C. rivularis* (*Cardamine* × *rhodopaea*) was described from the Rhodope Mountains in Bulgaria (Ančev et al., 2013). We collected 91 plants from nine populations, which also included putative hybrids with *C. matthioli*.

Altogether, we sampled 145 populations, which were used in flow cytometric (1,443 individuals) and microsatellite analyses (1,104 individuals). Chromosome numbers were counted from 58 populations (219 plants). A selection of 48 accessions, representing the observed overall taxonomic, geographic, ecological, and cytotypic variation, was subjected to Hyb-Seq analyses (Supplementary Data 1 and Figure 1).

### Chromosome Counting and Estimation of Relative Genome Size

Mitotic chromosomes were counted from young anthers or root tips fixed in an ethanol:acetic acid (3:1) mixture and stored in 70% ethanol at −20°C. Chromosome spreads were prepared following Marhold et al. (2002) or Mandáková et al. (2019). Chromosomes were stained using 4′,6-diamidino-2-phenylindole (2 mg/ml DAPI in Vectashield antifade; fluorescence signals were analyzed and photographed using a Zeiss Axioimager epifluorescence microscope with a CoolCube camera by MetaSystems) or using the Giemsa stain.

Relative nuclear DNA content was measured from fresh leaf tissues by flow cytometry (FCM) using the AT-selective DAPI fluorochrome (Doležel et al., 2007a, b). Each individual was analyzed separately or up to three individuals were pooled prior to measurements. Due to a wide range of ploidy levels and expected DNA content in the studied species, three internal standards with different 2C values were used: *Solanum lycopersicum* ‘Stupické polní rané’ (2C = 1.96 pg; Doležel et al., 1992), *Solanum pseudocapsicum* (2C = 2.59 pg; Temsch et al., 2010), and *Bellis perennis* (2C = 3.38 pg; Schönswetter et al., 2007). The DNA content of *Bellis* and *S. pseudocapsicum* was calibrated against *Solanum lycopersicum* based on three repeated analyses performed on different days. DNA ploidy level was inferred on the basis of DNA content measured in plants with counted chromosome numbers. Sample preparation followed the two-step procedure using Otto buffers (Doležel et al., 2007a, b) and the protocol described by Španiel et al. (2011). The fluorescence intensity of 5,000 particles (stained nuclei) was measured using a Partec CyFlow ML flow cytometer (Partec GmbH, Münster, Germany). The resulting flow cytometric histograms were evaluated using Partec FloMax software (v. 2.7d; Partec GmbH). Relative nuclear DNA content (relative genome size, 2C value given in arbitrary units; a.u.) was determined from the ratio between the positions of the G1 peaks of the sample and the standard. The coefficients of variation (CV) were calculated for both the internal standard and the sample to assess the reliability of measurements. Only histograms with CV values below the 5% threshold were accepted.

### Amplification and Scoring of Microsatellite Loci

Total genomic DNA was extracted from silica gel-dried young leaves using the DNeasy Plant Kit (Qiagen, Hilden, Germany).

The microsatellite markers used in this study were developed from genomic sequence data of two individuals of *Cardamine amara* and *C. pratensis* (an individual referred to as *C. rivularis* auct.) following Zozomová-Lihová et al. (2014a). Out of 50 SSR loci with di-, tri-, and tetranucleotide repeat motifs tested for amplification and allelic variation, 18 loci (Supplementary Table 1) proved successful and were used for the final analyses. Amplifications were performed in five multiplex assays using the Multiplex PCR kit (Qiagen) and fluorescently labeled primers following the protocols detailed in Zozomová-Lihová et al. (2014a). The Applied Biosystems 3130xl Genetic Analyzer (DNA Sequencing Laboratory, Faculty of Science, Charles University, Prague, Czechia) was used to separate and visualize the alleles. To check the consistency of amplification and allele determination, 86 replicate samples were included.

Allele sizes were recorded using Geneious R7.1 and R10 software (Kearse et al., 2012) with the microsatellite plugin version 1.4.4. Taking into account the high ploidy level of many samples (up to 7*x*), we did not estimate the allele copy number in partial heterozygotes from peak heights, as described in Esselink et al. (2004). Alleles were recorded as either present in or absent from each sample, regardless of the number of allele copies, inferring ‘marker phenotypes’ or ‘allelic phenotypes’ (following the terminology by Meirmans and van Tienderen, 2004; Dufresne et al., 2014; Meirmans et al., 2018) instead of complete genotypes.

### Target Enrichment Probe Design

Probes targeting orthologous low-copy nuclear loci were developed using Sondovač 0.99^1^ (Schmickl et al., 2016), which is based on a combination of transcriptome and genome skim data. For the input, paired-end genome skim raw data of *Cardamine parviflora* (NCBI accession number: SRR11977919), a mitochondrial sequence of *Arabidopsis thaliana* (NCBI accession number: NC_001284.2), a plastome sequence of *C. impatiens* (NCBI accession number: KJ136821.1), and transcriptome sequences of *C. amara* (NCBI accession number: SRR11977918) were used. These sequences of *C. parviflora* and *C. amara* were generated as part of this study on the HiSeq2500 Illumina platform. The transcriptome of *C. amara* was assembled using Trinity 2.0.6 (Grabherr et al., 2011). Sondovač was executed with the above-mentioned input data, using default values, except for the ‘Maximum overlap length expected in approximately ≥ 90% of read pairs’ that was set to 200. Transcriptome sequences that were at least 90% similar and genome reads mapping to mitochondrial or chloroplast genomes were removed. Genome skim reads were matched to transcripts, filtered, and *de novo* assembled in Geneious 7.1.9 (Kearse et al., 2012). Assembled contigs were filtered for length (retaining contigs >120 bp, total length of all contigs for a transcript >1,200 bp) and uniqueness (with a threshold value of 90%). Sequences sharing more than 90% similarity with the plastome sequence were removed. Retained contigs were compiled as target sequences for probe synthesis. In total, 14,464 120-mer probes (biotinylated RNA baits) were synthesized by MYcroarray (now Arbor Biosciences, MI, United States), targeting 2,246 exons from 1,235 genes.

### Illumina Library Preparation for Hyb-Seq

Genomic DNA (400 ng per accession) was fragmented with Covaris S220 or M220 sonicators (Woburn, MA, United States) to a target fragment size of 500 bp. Sequencing libraries were prepared using the NEBNext^®^ Ultra^TM^ DNA Library Prep Kit for Illumina^®^ following the manufacturer’s protocol (New England Biolabs, MA, United States). After cleanup of adaptor-ligated DNA with the QIAquick PCR Purification Kit (Qiagen), size selection was performed by SPRIselect beads (Beckman Coulter, MA, United States) to produce 500–600 bp long fragments. Amplification was performed with 8 cycles of PCR, using index primers from NEBNext^®^ Multiplex Oligos for Illumina^®^. The amplified libraries were cleaned up with AMPure XP beads (Beckman Coulter) and pooled equimolarly (24 accessions/pool). The pooled libraries were enriched by hybridization with synthesized RNA baits (at 65°C for 26 h) using the MYbaits kit, following the protocol v. 3.02 (Arbor Biosciences). The target-enriched libraries were PCR amplified for nine cycles with the KAPA HiFi HotStart mix (Kapa Biosystems, Cape Town, South Africa), purified with the QIAquick PCR Purification Kit and submitted for sequencing with 150 bp paired end reads on an Illumina MiSeq system at BIOCEV or CEITEC, Czechia.

### Environmental Data

Environmental data were acquired for each of the sampling sites of the studied species, complemented by published records (Marhold, 1992, 1994a; Kučera et al., 2005; Ančev et al., 2013) that could be georeferenced and unequivocally attributed to the species and here recognized genetic lineages. Overall, 346 sites (see Supplementary Data 2) were included. The environmental data consisted of 72 habitat characteristics (Supplementary Data 2). Climatic and terrain variables were preprocessed by GeoModel Solar (Bratislava, Slovakia), as described in detail by Zozomová-Lihová et al. (2015). The temperature data (30 arc-sec resolution) consisted of average air temperature values for the period 1990–2009 (Saha et al., 2010). The precipitation data (having 2 arc-min resolution) covered a period from 1951 to 2000 (Schneider et al., 2014). Solar radiation was calculated using the SolarGIS model (15 arc-sec resolution) based on monthly long-term averages (1994 to 2013). Global tilted irradiation represents the thermal regime of the soil and vegetation cover whereas photosynthetically active radiation quantifies radiation available for plant photosynthesis. A digital elevation model was derived from SRTM3 data (3 arc-sec resolution). We also added bioclimatic variables obtained from the WorldClim database at 30 arc-sec resolution (Fick and Hijmans, 2017).

### Data Analyses

#### Flow Cytometry

Differences in relative DNA content were tested in R version 3.3.2 (R Core Team, 2019) using the non-parametric Mann-Whitney test. The spatial segregation of cytotypes was tested using the Mantel test (Mantel, 1967) as implemented in the R package ade4 (Dray and Dufour, 2007). A pairwise distance matrix calculated from the geographic coordinates of populations was compared with a pairwise binary matrix of ploidy levels coding cytotypic identity. Mixed ploidy-level populations were excluded. Significance levels were estimated by random rearrangements (9,999 replicates) of the original matrices.

#### Microsatellite Datasets

Genotypic data from SSR loci were exported from Geneious in the GeneMapper format, which was loaded in the POLYSAT 1.5-0 package (Clark and Jasieniuk, 2011). The POLYSAT package was used for generating input data files for STRUCTURE (Pritchard et al., 2000) and GenoDive (Meirmans and van Tienderen, 2004), for creating partial data matrices (see below), and for calculating Lynch distance (Lynch, 1990) between individuals or lineages. This distance is a simple measure of band-sharing similarities, which best suits our data. The error rate was calculated from the binary matrix exported from POLYSAT, as the ratio of mismatches over matches of the alleles determined in the profiles of the duplicated samples. The following data matrices were assembled: (1) Complete Matrix: It consisted of all 1,104 samples, and was employed to infer the overall genetic structure, to identify potential interspecific hybrids, and to calculate population diversity parameters. (2) MajMatRiv Matrix: This partial matrix comprised *C. matthioli*, *C. majovskyi*, and *C. rivularis*. It was employed to elucidate the deeper structure within these species, and to identify potential gene flow and the occurrence of hybrids in the contact zone of *C. matthioli* and *C. rivularis* in Bulgaria. (3) Prat Matrix: This matrix comprised samples of *C. pratensis*, and was used to uncover the deeper structure within this species. The latter two data matrices corresponded to the clusters identified by STRUCTURE analyses (see below) of the Complete Matrix. Hybrid populations (see below for details on their identification) were generally omitted from the partial datasets but retained in cases when both parental clusters were present in the partial dataset.

#### Microsatellite Data Analyses

Because allele dosage could not be reliably estimated, standard population genetic diversity statistics that require genotype or allele frequencies (such as F_*ST*_ and expected heterozygosity, H_*E*_) could not be calculated (Bruvo et al., 2004; Obbard et al., 2006). Therefore, we used GenoDive 2.0b27 (Meirmans and van Tienderen, 2004) and POLYSAT 1.5-0 (Clark and Jasieniuk, 2011), which can handle genetic data from polyploids and mixed-ploidy datasets, and correct for unknown dosage of alleles in partial heterozygotes. Genetic diversity was evaluated through the following descriptive statistics: total number of alleles (A), number of population- and species-specific (private) alleles (priv.; both calculated manually), average number of alleles per locus (A′), effective number of alleles (Ae), total heterozygosity (H_*T*_), all three calculated in GenoDive, and Shannon diversity index (ShDI) calculated in POLYSAT. In addition, frequency down-weighted marker indices for individuals and populations (Rarity1, Rarity2; Schönswetter and Tribsch, 2005) were calculated using the R script AFLPdat (Ehrich, 2006). The diversity statistics for each lineage (or species) were calculated as the average of values obtained for populations, subjected to rarefaction correction to account for unequal number of populations. Only populations that unequivocally belonged to the lineage/species (omitting those identified as hybrid, see below) and with at least seven samples were included. In addition, allelic richness (α) was estimated for each species or lineage following the generalized rarefaction approach in ADZE v. 1.0 (Szpiech et al., 2008).

Bayesian clustering of individuals was performed to infer homogeneous genetic clusters and detect genetic admixture with STRUCTURE 2.3.4 (Pritchard et al., 2000). This model-based clustering has proven to provide unbiased inference from mixed-ploidy data with unknown allele dosage, even when population differentiation was weak (Stift et al., 2019). Ten replicates for each *K* = 1–10 were run with the settings and result processing as described in Zozomová-Lihová et al. (2014a). The approach of Evanno et al. (2005) was used to determine the optimal *K* value.

Hybrid identification followed the STRUCTURE results. As an operational unit we chose the level of population. Thus, the putatively hybrid populations were either kept or removed as a whole from the partial datasets and further analyses, instead of removing single, potentially hybrid individuals. A population was considered hybrid if genetic admixture of at least a half of its samples exceeded the threshold of 25%, and the geographic proximity of the relevant species or lineages also favored gene flow among them.

A neighbor-joining (NJ) tree was calculated using the package ape (Paradis et al., 2004) in R 3.3.2 (R Core Team, 2019). A pairwise distance matrix of individuals was calculated using Lynch distances (Lynch, 1990), as implemented in the R package POLYSAT. Trees were visualized using FigTree software version 1.4.4^2^. The genetic similarity between the taxa or lineages was also expressed by pairwise Lynch similarity indices. A hierarchical analysis of molecular variance (AMOVA) as described in Excoffier et al. (1992) was performed using the R package poppr 2.8.2 (Kamvar et al., 2014, 2015). The groups defined in this analysis followed the clustering results obtained with STRUCTURE and NJ tree. Potential hybrid populations, as described above, were omitted from the input matrices. The significance of estimated genetic partitioning was tested using 999 permutations.

#### Hyb-Seq Data Analyses

Demultiplexed reads were trimmed of adapters and quality-filtered using Trimmomatic-0.36 (Bolger et al., 2014). Bases at read ends with quality <Q20 were discarded, and the remaining part of the read was trimmed beyond read ends, if average quality in a 4 bp sliding window was <Q15. Finally, any reads trimmed to under 50 bp were discarded. PCR duplicates were removed using the Clumpify command of BBTools^3^.

Two approaches were employed for further data processing: the first was based on the assembled genes (consensus or allele sequences obtained by read-backed phasing), while the second one utilized single-nucleotide polymorphism (SNP) calling and Bayesian clustering of the SNP datasets.

Consensus sequences were assembled using HybPiper version 1.3 (Johnson et al., 2016). Reads were mapped to the target probe sequences using BWA (Li and Durbin, 2009), assembled into contigs using SPAdes (Bankevich et al., 2012), and coding sequences were identified using Exonerate (Slater and Birney, 2005). The ‘supercontigs’ (targeted exons and flanking sequences) were recovered using the script intronerate.py. The supercontig sequences were aligned with MAFFT (Katoh and Toh, 2008). Flanks and sites with gaps in more than 25% of sequences were removed using the trimEnds and deleteGaps functions of the ips package (Heibl, 2008 onward) in R 3.3.2 (R Core Team, 2019). Alignment statistics were calculated with AMAS (Borowiec, 2016), and alignments displaying extreme values in descriptive statistics were inspected visually; misassemblies were removed. Finally, 1,487 supercontigs which passed inspection were concatenated into 963 genes.

Maximum-likelihood gene trees were constructed for 499 genes containing at least 32 phylogenetically informative sites (calculated with AMAS; Borowiec, 2016) using RAxML-NG v. 0.9.0 (Kozlov et al., 2019). The best-fit model of substitution for each gene was estimated using the IQ-TREE’s ModelFinder function (Kalyaanamoorthy et al., 2017) under the Bayesian information criterion. Bootstrap analyses were performed using 1,000 replicates. Internal branches with bootstrap support ≤20% were collapsed with Newick-Utilities v. 1.6 (Junier and Zdobnov, 2010). To summarize the gene trees as well as to visualize potential incongruences among them, a supernetwork was inferred with SuperQ v.1.1 applying JOptimizer scaling and Gurobi optimizer (Grünewald et al., 2013; Bastkowski et al., 2018). In this analysis, the gene trees are decomposed into weighted quartets and then stitched together to a split network using the QNet algorithm (Grünewald et al., 2013).

Allele sequences were derived using the scripts and the workflow available at https://github.com/mossmatters/phyloscripts/tree/master/alleles_workflow, described in detail by Kates et al. (2018), but using the latest versions of GATK and WhatsHap (Martin et al., 2016) enabling to call and phase also polyploid variants. If the phased sequences were divided to multiple blocks, only the longest phase block for each individual was retained and the remaining variant sites were replaced with ambiguity characters. Phased alleles obtained from all 1,487 supercontigs were concatenated using AMAS (Borowiec, 2016) and a phylogenetic tree was constructed using the maximum likelihood approach after automatic model selection for each charset of a best-fit partitioning scheme using ModelFinder (Chernomor et al., 2016; Kalyaanamoorthy et al., 2017) and RAxML-NG v. 0.9.0 (Kozlov et al., 2019). Bipartition support was computed from 100 bootstrap replicates.

Bayesian clustering of the SNP datasets was obtained using STRUCTURE 2.3.4 (Pritchard et al., 2000). Variant calling and subsequent parsing to STRUCTURE format was performed using the snipStrup pipeline, which takes into account different ploidy levels of the analyzed samples (Šlenker et al., in prep.^4^). This pipeline uses BWA (Li and Durbin, 2009) to align reads to reference sequences, samtools (Li et al., 2009) for sorting and indexing, GATK (McKenna et al., 2010) for variant calling and filtering, and the R packages ape (Paradis et al., 2004) and vcfR (Knaus and Grünwald, 2016; Knaus and Grünwald, 2017) for allele extraction. As a reference, target sequences (used for probe synthesis) of 1,487 exons (those whose alignments passed inspection) were used. Exons were concatenated to genes and, to ensure no linkage existed between sites, 500 datasets were produced by drawing a single random SNP site from each gene. The datasets were piped to STRUCTURE 2.3.4 (Pritchard et al., 2000), which was run as with the microsatellite data above. The results of 500 analyses were averaged using the program CLUMPP (Jakobsson and Rosenberg, 2007) and drawn with distruct (Rosenberg, 2004). To uncover the deeper genetic structure within the species, SNP variation of *C. pratensis* (Prat dataset) and the remaining samples (MajMatRiv dataset) were analyzed separately.

#### Environmental Niche Differentiation

The environmental niches of the species under study and their genetic lineages were defined using 72 GIS-derived habitat characteristics (Supplementary Data 2), rescaled to zero mean and unit standard deviation. To quantify differences in niche positions and niche breadths, the environmental heterogeneity was summarized in a matrix of standardized Euclidean distances among the sites.

We used redundancy analysis (RDA; Rao, 1964) to test for overall differences in habitat characteristics between species and genetic lineages (sites with equivocally classified, putatively hybrid populations were removed). Randomization tests with 10,000 unrestricted permutations were used to calculate probabilities. Results of RDAs were plotted in ordination space to visualize environmental niche shifts among the groups.

A matrix of standardized Euclidean distances was used for principal coordinate analysis (PCoA) and niche breadth was quantified using the dispersion of sites from their spatial median in ordination space. A distance-based test of homogeneity of multivariate dispersions (Anderson, 2006) was employed to compare environmental niche breadths among the species and genetic lineages. Probabilities for the test statistic *F*_*m*_ were calculated from 10,000 unrestricted permutations of the least-absolute-deviation residuals.

Finally, multinomial generalized linear models (GLMs; McCullagh and Nelder, 1989) were used to identify subsets of environmental variables accurately discriminating habitats of the defined groups. Because the environmental dataset was truly multidimensional (72 habitat characteristics) and many variables were strongly correlated, which would pose problems related to unstable estimates of coefficients in a standard GLM (Dormann et al., 2013), we used penalized GLMs with the least absolute shrinkage and selection operator penalty (LASSO; Tibshirani, 1996). The LASSO is a regularization technique shrinking the coefficients toward zero and able to set some coefficients exactly to zero, that is, to conduct variable selection. The amount of penalization is controlled by the regularization parameter λ; as the λ penalty becomes large, fewer coefficients are left in the model. Finding an optimal value of the regularization parameter is subject to tuning. We used a 10-fold cross-validation procedure to select the final model with the λ penalty yielding the lowest misclassification rate.

Analyses were performed in R (R Core Team, 2019) using the libraries caret (Kuhn, 2019), ggplot2 (Wickham, 2016), glmnet (Friedman et al., 2010), and vegan (Oksanen et al., 2019).

## Results

### Cytotypic Composition and Chromosome Counts

Chromosome numbers were counted for 219 plants from 58 populations (Supplementary Data 1 and Supplementary Figure 1). We found extensive variation in chromosome numbers, consisting of the counts of 2*n* = 16, 17, 18, 19, 20, 22, 28, ca. 28–30, 30, 31, 32, ca. 33, 34, 36, 37, 38, ca. 34–40, ca. 42–44, 44, 48, 52, which represent diploid, tetraploid and hexaploid cytotypes (with the base number *x* = 8) along with frequent occurrence of probable aneuploids and dysploids. Although the chromosomes did not differ significantly in size, the presence of B chromosomes cannot be ruled out either. In addition, triploids and heptaploids were inferred from relative DNA content (not confirmed by chromosome counting) and based on the number of alleles recorded per microsatellite locus.

**FIGURE 2:**
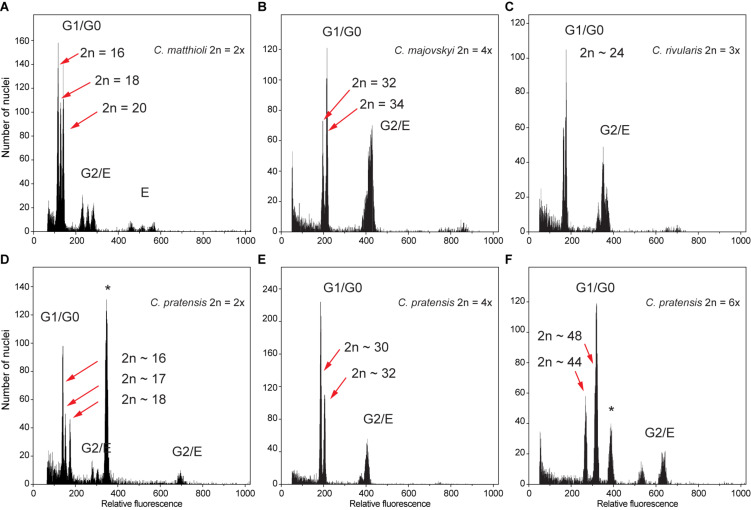
Flow cytometric histograms of DAPI-stained nuclei obtained from simultaneous analyses of multiple samples from the same populations, documenting intraspecific divergence in nuclear DNA content in the *Cardamine* species under study caused by both aneuploidy and dysploidy. **(A)** Diploid *C. matthioli*, population Mat_CAJ; **(B)** tetraploid *C. majovskyi*, population Maj_KIS; **(C)** triploid *C. rivularis*, population Riv_KAT; **(D)** diploid *C. pratensis*, population Prat_DE5; **(E)** tetraploid *C. pratensis*, population Prat_AIS; **(F)** hexaploid *C. pratensis*, population Prat_GOR. Letters denote peaks of nuclei corresponding to different phases of the cell cycle (G0–G2) and/or levels of endopolyploidy **(E)**; the internal standards *Solanum pseudocapsicum* and *Bellis perennis* used in analyses **(D,F)**, respectively, are marked by an asterisk. For locality codes see Supplementary Data 1.

Flow cytometry analyses mostly yielded high-resolution histograms, with an average CV for the samples of 2.09% (range 1.1–4.9%) and 1.89% (range 0.94–4.62%) for the standards. In several populations, some individuals showed minor differences in their relative 2C values, which was most likely caused by aneuploidy and dysploidy (indeed, in a number of cases confirmed by direct chromosome counting, see Supplementary Data 1). Simultaneous flow cytometric analyses of such individuals yielded histograms with clearly separated or bifurcated peaks and confirmed that these differences reflected real variation (Figure 2).

#### Cardamine matthioli

**FIGURE 3:**
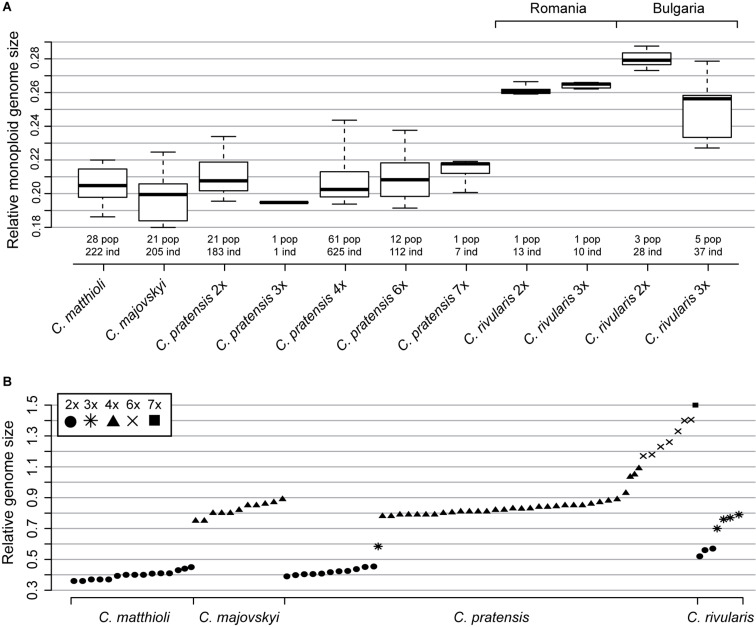
Genome size variation among and within *Cardamine matthioli*, *C. majovskyi*, *C. pratensis*, and *C. rivularis.* **(A)** Relative monoploid genome size (C*x* value, following the terminology of Greilhuber et al., 2005) of the species and cytotypes studied. Boxes show interquartile range, and whiskers are extended to the 5th and 95th percentiles. **(B)** Relative genome size (2C) of the species under study (population mean values). Only representative populations are plotted. The fluorescence intensity of the internal standard *Solanum lycopersicum* (2C = 1.96 pg) was set to a unit value. Values given under *C. rivularis* 3*x* from Bulgaria most likely include both autotriploids of *C. rivularis* and hybrids between *C. rivularis* and *C. matthioli* (see the text for further explanations). Detailed population-, cytotype-, and species-level values are listed in Supplementary Data 1 and Supplementary Table 2.

All samples of *C. matthioli* analyzed for this study were confirmed to be diploid. Nevertheless, extensive intraspecific variation in nuclear DNA content was found, which exceeded 40% (Supplementary Table 2 and Figure 3). At the level of populations, the variation ranged from negligible to as much as 37.58%, which was caused either by aneuploidy or the presence of B chromosomes (2*n* = 16–20, 22; Supplementary Data 1 and Supplementary Figure 1). Genome sizes derived from population averages for the two lineages defined by genetic data (denoted as the Central and the Widespread lineage, see below) did not differ significantly (Mann-Whitney test, *Z* = −1.47, *p* = 0.142).

#### Cardamine majovskyi

All analyzed samples of *C. majovskyi* were tetraploid. Intraspecific variation in nuclear DNA content reached 22.65% (omitting the potentially hybrid population Maj_LOG, see below), which is, as in the case of *C. matthioli*, attributable to aneuploidy (2*n* = 32, 34; Supplementary Data 1 and Supplementary Figure 1). The intrapopulational variation reached up to 12.34%. Similarly as in *C. matthioli*, relative genome sizes of *C. majovskyi* did not differ significantly between the Central and the Widespread lineage (*Z* = −0.05, *p* = 0.96). In the population Maj_LOG, where plants with 2*n* = 31 and 2*n* = mosaic of 36 and 40 were recorded (Supplementary Figure 1), and where hybridization with *C. pratensis* is assumed, the extent of intrapopulational variation was 22.24% (Supplementary Data 1).

#### Cardamine rivularis

Samples of *C. rivularis* (and its putative hybrids with *C. matthioli*) exhibited large variation in relative DNA content (Figure 3 and Supplementary Figure 2). Although precise chromosome numbers were not determined in the present study (due to the lack of living material), chromosome number records were previously published from the same populations as examined here (Marhold, 1994a; Ančev et al., 2013). Thus, the DNA content measured herein could be unequivocally attributed to two ploidy levels as previously published, diploids and triploids. Different variation patterns were observed in Romanian and Bulgarian populations, the latter being affected by hybridization with *C. matthioli* (see below).

The two Romanian populations of *C. rivularis* analyzed were cytologically uniform, one consisting of diploids and the other of triploids. The variation in relative DNA content in both populations was less than 5%, and their monoploid relative genome sizes were almost identical (C*x* = 0.262 and 0.264 for 2*x* and 3*x*, respectively; Supplementary Table 2).

Much greater variation was revealed in Bulgarian populations. Diploids were recorded in three out of the seven analyzed populations, and their genome size variation was relatively low (7.09%). Their monoploid genome size, however, was larger than that of diploids from Romania (C*x* = 0.28 in Bulgarian vs. C*x* = 0.262 in Romanian diploids, Supplementary Table 2 and Figure 3A). The rest of the samples, putatively assigned to triploids, exhibited large variation in genome size (2C = 0.681–0.865; 27.06%; Figure 3). Individuals with the highest 2C values (2C ∼ 0.84; Supplementary Figure 2B) may represent triploids of *C. rivularis* because their monoploid genome size corresponds to that of sympatric diploids. On the other hand, the triploids with the lower 2C values most likely represent hybrids with *C. matthioli* (which has significantly smaller monoploid genome size than diploid *C. rivularis*; Supplementary Table 2 and Figure 3A), where the unreduced gamete was provided either by *C. matthioli* (expected mean 2C ∼ 0.678) or *C. rivularis* (expected mean 2C ∼ 0.759). The co-occurrence of diploids with triploids was observed in one population, whereas in four populations, only triploids were recorded (Supplementary Data 1). The number of plants analyzed per population, however, was low (8–10), so we assume that diploid-triploid co-occurrence within populations may actually be more frequent.

#### Cardamine pratensis

The analyzed samples of *C. pratensis* comprised several cytotypes ranging from diploid to heptaploid. The majority of individuals were tetraploids (approximately 67%) whereas diploids and hexaploids made up 20 and 12% of the samples, respectively (although the assignment of some aneuploid individuals to ploidy levels might be equivocal). In addition, two rare odd-ploidy level cytotypes were inferred from FCM analyses: one heptaploid population and a single triploid individual in an otherwise diploid population. Almost 90% of the populations sampled consisted exclusively of one ploidy level. Sympatric occurrences of two cytotypes were encountered in only nine populations (Supplementary Data 1).

The distribution of ploidy levels in Central Europe departed from a random pattern (Mantel *r* = 0.23, *p* < 0.001). Diploid populations occupied three geographically distinct areas: in the Eastern Carpathians, the Alps, and central and southern Germany. The distribution of tetraploids overlapped with that of diploids and in general covered the whole distributional range of *C. pratensis*. Hexaploid populations were predominantly found in the Western Carpathians and adjacent areas. Several spatially isolated hexaploid samples were found in four mixed-ploidy populations in Switzerland, Slovenia, and Lower Austria. The single heptaploid population was recorded in Styria, Austria (Supplementary Data 1 and Figure 1).

The intra-cytotypic genome size variation amounted to 35.54% in diploids, 50.37% in tetraploids, and 29.95% in hexaploids (Supplementary Table 2). This large variation in nuclear DNA content is congruent with the detected spectrum of chromosome numbers (i.e., aneuploidy and dysploidy). Monoploid relative DNA content of dominant cytotypes was almost identical among all main ploidy levels (C*x* = 0.21, 0.208, and 0.211 for 2*x*, 4*x*, and 6*x*; Figure 3).

Within the diploid cytotype, chromosome numbers ranged from 2*n* = 16 to 19. At the tetraploid level, they varied from 2*n* = 28 to 38, reflecting most likely both aneuploidy and dysploidy. Apart from supernumerary chromosomes counted sporadically (i.e., aneuploidy), tetraploid *C. pratensis* possessed two cytotypes: regular tetraploid (2*n* = 4*x* = 32) and hypotetraploid caused by chromosome fusion (dysploidy, 2*n* = 4*x* = 30). Both cytotypes are widely distributed across the sampled range. At the hexaploid level, 2*n* = ca. 42–44, 44, 48, 52 chromosomes were confirmed (Supplementary Figure 1). Similarly to the case of tetraploids, hexaploids comprise two main cytotypes, of which the hypohexaploid one (dysploidy, 2*n* = 6*x* = 44) is more common than the regular hexaploid one (2*n* = 6*x* = 48; Supplementary Data 1).

Relative genome size values (2C) of diploid *C. matthioli* overlap with those of diploids of *C. pratensis*, as do also 2C values of tetraploid *C. majovskyi* with the tetraploids of *C. pratensis* (Figure 3A, Supplementary Figure 2, and Supplementary Table 2). By contrast, diploids of *C. rivularis* (both from Bulgaria and Romania) had markedly larger genome size than *C. matthioli* and diploid *C. pratensis*.

### Genetic Structure Based on Microsatellite Variation

The 18 microsatellite loci used yielded 394 alleles. The total number of alleles and phenotypes detected per locus ranged significantly, from five alleles (eight ‘marker phenotypes’) in the locus Card12 to 58 alleles (528 ‘marker phenotypes’) in the locus Card19 (Supplementary Table 1). The error rate calculated from the replicates was 0.001 on average, and ranged from 0 to 0.02.

#### Complete Matrix

**FIGURE 4:**
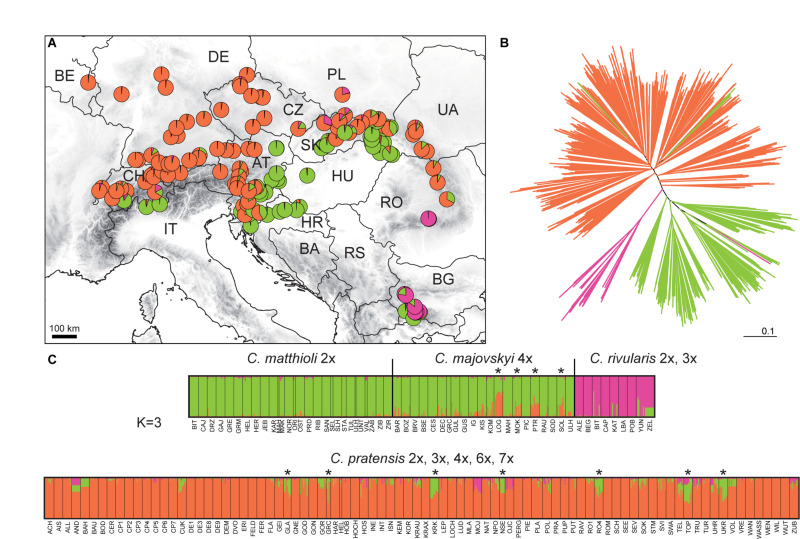
Genetic structure of all species studied, *Cardamine matthioli*, *C. majovskyi*, *C. rivularis*, and *C. pratensis*, as inferred from microsatellite data analyses of the Complete Matrix. **(A)** Distribution of the sample sites. Different colors show the population assignment to three genetic clusters as inferred from the STRUCTURE analyses. **(B)** Neighbor-joining tree diagram, calculated using Lynch distances. **(C)** Bayesian model-based clustering (STRUCTURE) at *K* = 3. The coloring of vertical bars indicates each individual’s proportional cluster assignment: green: *C. matthioli – C. majovskyi*; purple: *C. rivularis*; orange: *C. pratensis*. Putative hybrid populations between *C. majovskyi* and *C. pratensis* are marked by asterisks. For locality codes see Supplementary Data 1.

The STRUCTURE analysis of the Complete Matrix comprising all 1,104 accessions and 394 alleles showed the following optimal genetic partitioning into three clusters (Figures 4A,C): (1) populations of *C. matthioli* and *C. majovskyi*, (2) *C. rivularis*, and (3) *C. pratensis*. The neighbor-joining clustering was consistent with this structure (Figure 4B). AMOVA suggested that 18.34% of genetic variation can be explained by differences between these three clusters, 31.1% by differences between populations, and 50.56% of the variation was present within populations (Table 2). The pairwise Lynch similarity index showed that the clusters *C. pratensis* and *C. matthioli – C. majovskyi* are genetically the most similar (74.83%) and that *C. rivularis* is genetically closer to *C. matthioli – C. majovskyi* than to *C. pratensis* (58% vs. 49.41%, respectively).

**TABLE 2:** Results of AMOVA demonstrating partitioning of genetic variation based on microsatellite markers.

Source of variation	d. f.	Sum of squares	Variance components	Percentage of variation	Φ – statistics
**(A) *Cardamine pratensis* complex**					
Between clusters	2	2725.494	4.817	18.34	ΦCT = 0.183***
Between populations within clusters	133	10070.431	8.171	31.1	ΦSC = 0.381***
Within populations	904	12009.773	13.285	50.56	ΦST = 0.494***
**(B) Genetic lineages of *C. matthioli* and *C. majovskyi***					
Between lineages	1	460.168	2.626	14.81	ΦCT = 0.148***
Between populations within lineages	43	1947.602	4.909	27.7	ΦSC = 0.325***
Within populations	278	2832.936	10.19	57.49	ΦST = 0.425***
**(C) Genetic lineages of *C. pratensis***					
Between lineages	2	885.793	2.634	10.37	ΦCT = 0.104***
Between populations within lineages	53	4390.685	7.846	30.87	ΦSC = 0.344***
Within populations	388	6195.837	14.93	58.76	ΦST = 0.412***

***p-value < 0.001. (A) Between genetic clusters identified within the Cardamine pratensis complex (C. matthioli + C. majovskyi, C. pratensis, C. rivularis), (B) between genetic lineages identified within the C. matthioli + C. majovskyi cluster (Widespread and Central lineages), and (C) between genetic lineages identified within C. pratensis (Red, Orange, and Yellow lineages). The delimitation of genetic clusters and lineages follows the results of STRUCTURE analyses.

This clustering corresponded to the taxonomic classification; however, individuals in several populations were assigned to more than one group, indicating potential gene flow between taxa. Applying the above defined criterion to the determination of hybrids (i.e., the threshold of 25% genetic admixture in at least half of individuals and geographic proximity), hybrid origins were indicated for 11 populations: four populations of *C. majovskyi* and seven populations of *C. pratensis* from the contact zone between these two species (marked by asterisks, see Figure 4C). In addition, several samples of *C. rivularis* showed genetic admixture of *C. matthioli*; however, these did not exceed half of the population sample size.

#### Partial Matrix of *C. matthioli*, *C. majovskyi*, and *C. rivularis*

**FIGURE 5:**
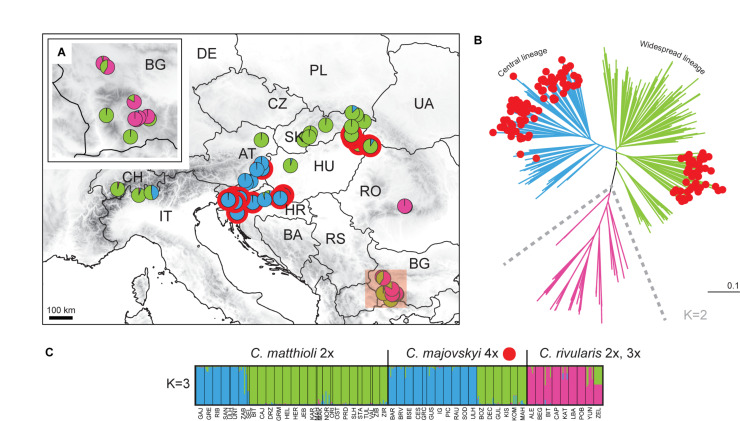
Genetic structure of *Cardamine matthioli*, *C. majovskyi*, and *C. rivularis* inferred from microsatellite data analyses of the MajMatRiv Matrix. **(A)** Distribution of the sample sites. Different colors indicate the population assignment to the three genetic clusters as inferred from STRUCTURE analyses. Circles with red outlines represent populations of *C. majovskyi.* **(B)** Neighbor-joining tree diagram, calculated using Lynch distances. Red dots highlight individuals of *C. majovskyi*. **(C)** Bayesian model-based clustering (STRUCTURE) at *K* = 3. Coloring of vertical bars indicates each individual’s proportional cluster assignment: blue: Central lineage; green: Widespread lineage; purple: *C. rivularis*. For locality codes see Supplementary Data 1.

The two genetic clusters of *C. rivularis* and *C. matthioli* with *C. majovskyi* (see above) were merged into a single dataset (MajMatRiv Matrix) comprising 389 samples from 54 populations and 254 alleles. STRUCTURE analysis of this matrix at *K* = 2 resulted in the same clustering pattern (results not shown) as the analysis of the complete matrix above. With increasing *K* to its optimal value (*K* = 3), individuals of *C. matthioli* and *C. majovskyi* were separated into two clusters irrespective of their ploidy level (and thus also taxonomy), which are clearly differentiated also in the NJ tree. These two lineages (denoted as Central and Widespread) showed a non-random spatial structure. The Widespread lineage is widely distributed across the species’ distribution range, including its western areas (border area of Italy and Switzerland), northern areas (Lower Austria, Slovakia, and northern Hungary) and southeastern areas (Bulgaria). By contrast, the Central lineage is localized in a relatively small area of Slovenia, northern Croatia, extending also to southeastern Austria (Styria and Burgenland) and western Hungary (Figure 5).

AMOVA revealed that the differentiation between the Central and the Widespread lineage explained 14.81% of variance (Table 2), while most variation is present within populations (57.49%), and the rest among populations (27.7%).

Populations belonging to the *C. rivularis* cluster are genetically different from the grouping of *C. matthioli* – *C*. *majovskyi*; however, gene flow between *C. rivularis* and *C. matthioli* in their contact zone is clearly manifested. Four populations of *C. rivularis* from Bulgaria included some individuals showing genetic admixture with the *C. matthioli* – *C. majovskyi* clusters (see Figure 5C). There was, however, no simple relationship between genetic admixture and ploidy level. Genetically admixed individuals were all triploid, but not all triploids exhibited signs of genetic admixture.

#### Partial Matrix of *C. pratensis*

**FIGURE 6:**
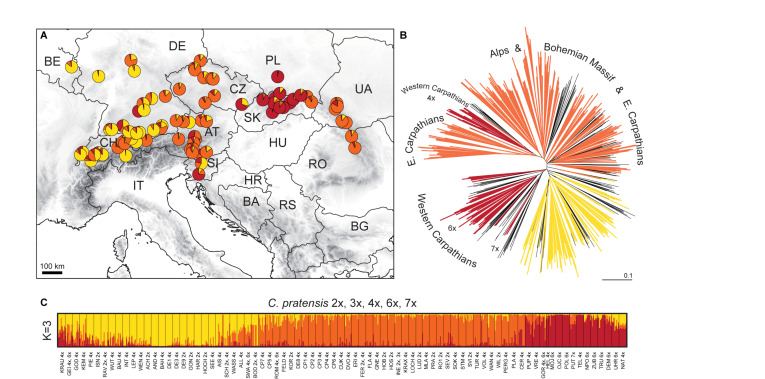
Genetic structure of *Cardamine pratensis* inferred from microsatellite data analyses of the Prat Matrix. **(A)** Distribution of sample sites. Different colors indicate the population assignment to three genetic clusters as inferred from STRUCTURE analyses. **(B)** Neighbor-joining tree diagram based on Lynch distances. Branches are colored according to the population assignment to three genetic clusters as inferred from STRUCTURE analyses; populations with equivocal (admixed) assignments are in black. **(C)** Bayesian model-based clustering (STRUCTURE) at *K* = 3. The coloring of vertical bars indicates each individual’s proportional cluster assignment: yellow: Western lineage; orange: Alpine-Bohemian-E Carpathian lineage; red: W Carpathian lineage. The ploidy level of each population is also indicated. For locality codes see Supplementary Data 1.

The partial matrix of *C. pratensis* (Prat matrix) comprised 635 samples from 80 populations and 368 alleles. STRUCTURE analyses indicated that a division into three clusters best explained the genetic structure of this dataset (Figure 6). The three lineages are spatially segregated in the longitudinal direction, and these are denoted here as the Western (‘Yellow’), Alpine-Bohemian-E Carpathian (‘Orange’), and the W Carpathian (‘Red’) lineage; for simplicity in the following text we refer to them by the colors as depicted in Figure 6. The Yellow lineage (Western) extends from Belgium and the lowlands of Germany to the Swiss Alps, and encompasses diploid and tetraploid cytotypes (plus a single hexaploid plant in one tetraploid population). Diploids of this lineage span over a wide elevational range, from foothills of the Alps (ca. 500 m) to alpine meadows (ca. 1,650 m). Tetraploids of this lineage are common in lowlands north of the Alps and in the montane zone (below 1,000 m), and only exceptionally reach higher elevations. Populations of the Yellow lineage partially overlap with those of the Orange lineage. The Orange lineage (Alpine-Bohemian-E Carpathian) comprises diploids and tetraploids occurring both in the Western and Eastern Alps, and expanding northwards to lower elevations of Austria, Germany (mainly Eastern Bavaria), and Czechia. This lineage also encompasses spatially isolated populations from the Eastern Carpathians and surrounding lowland areas. Diploids of the Orange lineage occupy montane and subalpine zones (ca. 900–1,900 m) in the Alps, while in the Eastern Carpathians they span from lowlands (350 m) up to the subalpine zone (ca. 1,900 m). Tetraploids of this lineage are dominant across Germany, Austria, and Czechia, and only scarcely occur in the Eastern Carpathians. They grow in lowland and montane belts, and, in contrast to tetraploids of the Yellow lineage, they also occupy (sub)alpine environment (ca. 1,000–1,800 m). Finally, the Red lineage (W Carpathian) occurs predominantly in the Western Carpathians and southern Poland. It encompasses tetraploids, hexaploids and one heptaploid population. The hexaploid cytotype is the most common and occupies the whole elevational gradient in this lineage (300–1,300 m). On the other hand, tetraploid and heptaploid populations are restricted to a narrow elevational range, from 600 to 800 m.

Nevertheless, considerable admixture among the three above-described lineages of *C. pratensis* is indicated by our STRUCTURE analyses (Figure 6C). The NJ clustering, too, indicates that genetic differentiation among the three lineages is weak and, in addition, suggests a split within the Red lineage (following the cytotypes) and subdivision within the Orange lineage (Figure 6B). AMOVA showed that only 10.37% of the variation was attributable to differences between the three lineages, and the majority of variation occurred within population (58.76%) (Table 2).

Populations previously classified as *C. nemorosa* and *C. udicola* (see Table 1 and Supplementary Data 1) are both included, among other populations, within the Yellow lineage. Not even the subalpine populations from the Alps and Eastern Carpathians, commonly referred to as *C. rivularis* auct., were identified by our analyses as a distinct entity, as they are placed both within the Yellow and the Orange lineage along with adjacent lowland populations. The morphotype ‘ucranica,’ reported from the foothills of the Eastern Carpathians (Table 1 and Supplementary Data 1), is placed within the Orange lineage.

### Genetic Diversity

Genetic diversity within the populations analyzed varied largely (Supplementary Data 1). After rarefaction correction, the highest diversity was recorded in *C. pratensis* and the lowest in *C. rivularis*. At the diploid level, diploid populations of *C. pratensis* were more diverse than those of *C. matthioli.* Similarly, at the tetraploid level, tetraploid populations of *C. pratensis* appeared more diverse than *C. majovskyi* (Table 3).

**TABLE 3:** Indices of genetic diversity based on microsatellite markers in the species, lineages, and cytotypes of the *Cardamine pratensis* complex.

Group	*N*_*ind*_	*N*_*pop*_	*A*	Priv.	*A*′	Ae	*H*_*T*_	Shan. div. ± SD	α ± *SE*
***Cardamine pratensis* complex**									
*C. matthioli + C. majovskyi*	296	37	228	12	3.293	2.311	0.496	1.01 ± 0.083	7.484 ± 0.665
*C. pratensis*	621	78	366	136	4.967	3.232	0.635	1.262 ± 0.118	10.786 ± 1.282
*C. rivularis*	72	9	119	9	2.321	1.835	0.368	0.477 ± 0	5.919 ± 0.705
**Diploids**									
*C. matthioli*	167	21	153	25	2.709	2.01	0.429	0.865 ± 0.117	6.319 ± 0.640
*C. pratensis*	134	17	231	92	3.419	2.403	0.53	1.051 ± 0.069	9.145 ± 1.242
*C. rivularis*	24	3	68	13	1.759	1.492	0.249	0.342 ± 0	3.778 ± 0.698
**Tetraploids**									
*C. majovskyi*	129	16	208	24	4.077	2.709	0.582	1.193 ± 0	7.560 ± 0.650
*C. pratensis*	361	45	320	136	5.174	3.294	0.648	1.298 ± 0.081	10.291 ± 1.228
**Hexaploid**									
*C. pratensis*	79	10	276	−	6.811	4.179	0.724	1.61 ± 0	10.335 ± 1.093
**Genetic lineages of *C. matthioli* and *C. majovskyi***									
Widespread	167	21	175	55	3.218	2.297	0.485	0.996 ± 0.037	6.827 ± 0.675
Central	129	16	173	53	3.413	2.333	0.507	1.031 ± 0	6.427 ± 0.576
**Genetic lineages of *C. pratensis***									
Yellow	128	16	231	14	4.206	2.858	0.58	1.037 ± 0.085	8.682 ± 1.046
Orange	247	31	294	41	4.557	2.943	0.611	1.248 ± 0.076	9.956 ± 1.235
Red	79	10	256	26	6.133	3.897	0.705	1.452 ± 0	10.088 ± 1.047

**N_*ind*_, number of analyzed individuals; N_*pop*_, number of populations included in the diversity calculations, i.e., without hybrid populations and those with low sample size (N_*ind*_ < 7). A, total number of alleles; priv., number of species- and lineage-specific (private) alleles; A′, average number of alleles per locus; Ae, effective number of alleles; H_*T*_, total heterozygosity; Shan. div., Shannon’s diversity index; α, allelic richness estimated with generalized rarefaction, calculated as the number of distinct alleles expected in a random subsample of size g, here g = 60. Values in A′, Ae, H_*T*_, and Shan. div. are population averages after rarefaction correction; α are values calculated at the species or lineage levels*.*

In *C. matthioli*, patterns of genetic diversity exhibited a non-random spatial structure. The highest values of the Shannon diversity and population rarity were detected in populations sampled in Slovenia, Croatia, and Slovakia (i.e., from both the Central and Widespread lineages) whereas peripheral Bulgarian populations appeared genetically depauperate (Supplementary Data 1). Swiss and Italian populations were omitted due to low sample sizes. When comparing the two lineages (comprising both *C. matthioli* and *C. majovskyi*), they displayed very similar genetic diversity values; the Central lineage appeared more diverse at the level of populations, but the overall estimated allelic richness was slightly higher in the Widespread lineage (Table 3). The percentage of genetic similarity between the two lineages was 72.16%.

In *C. pratensis*, the Yellow lineage was the least diverse, whereas the Red lineage was the most diverse, as shown by all parameters (Table 3). Some spatial structure in the diversity distribution was observed also within the lineages; however, most of this structure could be attributed to the distribution of different cytotypes, as is obvious from the positive correlation between ploidy level and detected diversity (Supplementary Data 1). Parameters of genetic rarity indicated high values especially in the Alps and Carpathians (the Red lineage and southern margins of both the Yellow and Orange lineages), while lower values were concentrated in northern regions of both the Yellow and Orange lineages (Supplementary Data 1). The three lineages showed high genetic similarity (pairwise Lynch similarity index), in congruence with their spatial distribution. Geographically overlapping lineages, the Orange and Red (82.76% similarity) and the Orange and Yellow (81.83% similarity) were less divergent than the geographically separated Yellow and Red lineages (78.85% similarity).

### Genetic Structure Based on Hyb-Seq

Sequencing process yielded, on average, 1.25 million reads per sample. Adapter trimming, quality filtering, and deduplication resulted in an average loss of 15.5% of reads. Of the remaining reads, 79.46% on average were mapped to target sequences. Out of the 2,246 exons from 1,235 genes, targeted by the designed RNA baits, 1,868 (82.8%) exons were recovered from all 48 samples. After inspection, 1,487 supercontig sequences were kept and concatenated to 963 genes of the overall length of 1,186,798 bases. The gene length ranged from 110 to 10,705 bp, with a mean of 1,232 bases. The proportion of parsimony informative sites ranged from 0.5 to 22.7%, with a mean of 3.8%. The variant calling approach (the snipStrup pipeline) utilized 936 genes (out of 963), which contained at least one variant for each sample. The genes harbored between one and 231 SNPs (omitting SNPs that occurred only in one sample), with an average of 41.21 and 47.86 variants per gene for the MajMatRiv and the Prat datasets, respectively.

**FIGURE 7:**
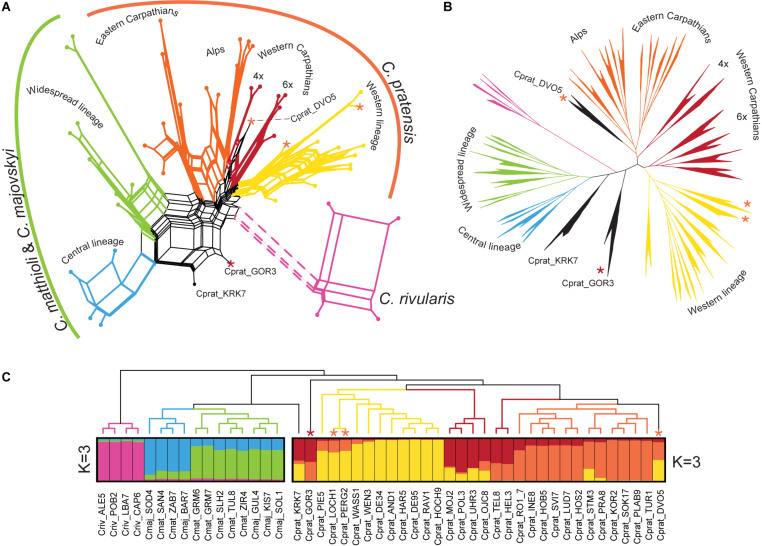
Genetic structure of *Cardamine matthioli*, *C. majovskyi, C. rivularis*, and *C. pratensis* inferred from Hyb-Seq data analyses. The coloring indicates the sample assignment to genetic clusters as inferred from STRUCTURE analyses: blue and green represent the Central and Widespread lineages of *C. matthioli* and *C. majovskyi*, respectively; purple represents *C. rivularis*; yellow, orange, and red represent the Western, Alpine-Bohemian–E Carpathian and W Carpathian lineages of *C. pratensis*, respectively. **(A)** Supernetwork representation of quartets generated in SuperQ, derived from 499 most informative maximum likelihood (ML) gene trees. **(B)** ML tree constructed from concatenated allele sequences obtained by read-backed phasing from all 963 genes in RAxML-NG. All major clades received bootstrap support greater than 95%. **(C)** Bayesian model-based clustering (STRUCTURE) based on SNP data from two partial datasets of *C. matthioli*, *C. majovskyi, C. rivularis* (on the left), and *C. pratensis* (on the right); ML tree constructed using the concatenated genes (as in **B**) is displayed as a cladogram on top. Samples with significant admixture (hybrid origins) between the species or lineages are shown with black branches. Asterisks denote samples differently placed in microsatellite analyses, and their colors indicate the assignment based on microsatellite data. For accession codes see Supplementary Data 1.

The different approaches that we employed for the Hyb-Seq data analyses, an ML tree based on phased allele sequences of all genes in concatenation, a supernetwork based on the most informative genes, and STRUCTURE analyses of SNP data drawn from the genes, provided highly congruent results (Figure 7). *C. rivularis*, *C. pratensis*, and *C. matthioli* intermingled with *C. majovskyi*, were identified as genetically clearly distinct. The extensive intraspecific variation observed within the latter three species is geographically structured, which is largely concordant with the patterns inferred from microsatellites. The same two genetic lineages, Central and Widespread, were identified within the clade of *C. matthioli–C. majovskyi*. Within *C. pratensis*, the same three lineages, denoted as Yellow, Orange, and Red, were resolved by STRUCTURE at *K* = 3 (Figure 7C), albeit with admixture observed especially within the Red lineage, besides some further subdivisions suggested by ML tree and supernetwork (Figures 7A,B). The Yellow lineage is most coherent, formed by western diploid and tetraploid populations from lowlands up to the subalpine belt in the Western Alps. Within the Orange lineage, the same differentiation between the Alpine range and the Eastern Carpathian range is indicated, both comprising populations from lowland to subalpine sites. The Red lineage from the Western Carpathians exhibits a subdivision based on ploidy levels; the hexaploid populations showed affinity to the Yellow lineage, whereas the tetraploids to the Orange one, which is also concordant with the microsatellite results. Only three populations from contact zones between the Yellow and Orange lineages were assigned differently by microsatellite and Hyb-Seq data (marked by asterisks, see Figure 7), which confirms the diffuse borders between these intraspecific lineages. Finally, two samples from Slovenia (Cprat_KRK7, Cprat_GOR3) were identified as potential hybrids between *C. pratensis* and *C. majovskyi* in the Hyb-Seq analysis, which was partly confirmed by microsatellite data (Figure 7). In conclusion, the Hyb-Seq data generated for a subset of 47 populations revealed a genetic structure that was highly congruent with that inferred from the microsatellite data of 145 populations.

### Environmental Niche Differentiation

**FIGURE 8:**
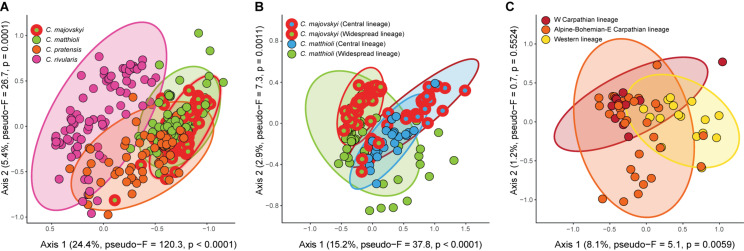
Results of RDA showing significant differences in the environmental niches of the four analyzed *Cardamine* species **(A)**, genetic lineages within *C. matthioli* and *C. majovskyi* **(B)**, and three genetic lineages within *C. pratensis* **(C)**. Ellipses define regions expected to contain 95% of all populations of the species or lineages. Vectors of habitat characteristics contributing to the ordination axes are shown in a full version of the RDA ordination graphs in Supplementary Figure 3. Variance explained by the ordination axes and results of randomization tests are given in parentheses. The scaling of the ordination plots is focused on habitat similarity among sites.

Redundancy analysis revealed significant differences in environmental niches of the four *Cardamine* species (pseudo-*F* = 50.5, *p* < 0.0001) (Figure 8A). The niches were separated along the main climatic gradient represented by elevation and accompanied changes in temperature and solar radiation. The niche of *C. rivularis* was typical for higher elevations, *C. pratensis* occupied milder mid-elevation sites, while *C. majovskyi* and *C. matthioli* occurred in lower elevations (Supplementary Data 2). The environmental niches of *C. matthioli* and *C. majovskyii* showed more overlap than the other species, but still differed, especially in the amount of photosynthetically active radiation and temperature seasonality (both higher in *C. majovskyi*). The multinomial LASSO model identified 42 habitat characteristics (Supplementary Table 3) that discriminate among species with relatively high overall predictive performance (79% of correctly classified species in cross-validation). The model showed excellent prediction of *C. rivularis* occurrence (0% misclassification). The rate of misclassification was greater in the case of *C. majovskyi* (28%), *C. matthioli* (29%), and *C. pratensis* (22%). The environmental niches of those species were of significantly different breadths (*F*_*m*_ = 18.7, *p* < 0.0001). *C. pratensis* and *C. rivularis* have broader niches than *C. majovskyi* (*p* = 0.0033 and *p* < 0.0001) and *C. matthioli* (*p* = 0.0004 and *p* < 0.0001). On the other hand, we found comparable niche breadths between *C. pratensis* and *C. rivularis* (*p* = 0.2541), and between *C. majovskyi* and *C. matthioli* (*p* = 0.9630).

The environmental niches of genetic lineages within *C. majovskyi* and *C. matthioli* (Central, Widespread, i.e., four groups in total), were significantly different (pseudo-*F* = 15.8, *p* < 0.0001) and each was statistically distinguishable from the others in pair-wise comparisons (Figure 8B). The main habitat-related difference was observed between the geographic areas along the precipitation gradient (greater precipitation sums in the more restricted Central region). Within the geographic areas, *C. majovskyi* typically occupies habitats with higher temperatures and lower seasonality of precipitation than *C. matthioli*. The final LASSO model consisted of 19 variables (Supplementary Table 3) which predicted the occurrence of the groups with good accuracy (the cross-validated overall classification accuracy equalled 78%). The classification error rate was quite high for *C. majovskyi* of the Central lineage (38%), but the other groups reached acceptable rates of misclassification (*C. matthioli*, Central – 12%; *C. majovskyi*, Widespread – 14%; *C. matthioli*, Widespread – 25%). We found the niche breadths of the four defined groups to be unequal (*F*_*m*_ = 4.1, *p* = 0.0075), but the only significant difference was between the two species within the Widespread lineage (*C. matthioli* > *C. majovskyi, p* = 0.01).

Genetic lineages within *C. pratensis* exhibited significantly different environmental niches (pseudo-*F* = 2.9, *p* = 0.0066). In the pairwise-comparison, habitat characteristics of the Yellow (Western) lineage differed significantly from both the Red and Orange lineages, while the latter two lineages were statistically indistinguishable (Figure 8C). In contrast to the Red and Orange lineages, the niche of the Yellow lineage is shifted toward more humid conditions with higher amounts of precipitation. The multinomial LASSO model involving 26 habitat characteristics (Supplementary Table 3) relatively accurately predicted the occurrence of the Yellow (25% cross-validated misclassification error rate) and Orange lineages (27%), however, the rate of misclassification was quite high in the Red lineage (46%). We found only marginally significant differences in niche breadths between the lineages (*F*_*m*_ = 3.3, *p* = 0.0436); the Orange lineage has a broader niche than the Red lineage (*p* = 0.0335), while the other pairs are statistically comparable.

## Discussion

### Patterns and Sources of High Cytotypic and Genome Size Variation

The results of the extensive flow cytometric screening of genome size and chromosome counting presented here support previous chromosome number reports (Kučera et al., 2005; Paule et al., 2017; Marhold et al., 2018) and describe the distribution patterns of ploidy levels and cytotypes in great detail. They underscore the extraordinarily large cytotypic variation within the *C*. *pratensis* complex in Central Europe, present both among and within the species. Apart from the euploid numbers, based on the primary base chromosome number *x* = 8, numerous aneuploids and dysploids were found. Aneuploidy was recorded in the diploid *C. matthioli*, tetraploid *C. majovskyi*, and across all ploidy levels of *C. pratensis*. In addition, descending dysploidy occurs at the tetraploid and higher levels in *C. pratensis.* The hypotetraploids with 2*n* = 30 possess one pair of markedly longer chromosomes, which were noticed already by Lawrence (1931) and Lövkvist (1956). Comparative chromosome painting revealed that the two long chromosomes underwent a nested chromosome insertion (fusion), a translocation event involving the ‘insertion’ of one chromosome into the pericentromere of the second chromosome, resulting in a chromosome number reduction from 2*n* = 32 to 30 (Mandáková et al., 2013). The hypotetraploids were most likely involved in the origin of hypohexaploid plants with 2*n* = 44 that were noted already by Lövkvist (1956) and detected also by the present study. Descending dysploidy, which has been recognized as a major route of diploidization of polyploid genomes and a significant mechanism of chromosome number evolution (Mandáková and Lysak, 2018; Udall et al., 2019), has been reported from several genera, including a pseudotriploid *Cardamine* species (Mandáková et al., 2016).

The co-occurrence of euploid and dysploid cytotypes in *C. pratensis*, along with aneuploids, may be facilitated by the perennial life-form and the capability for vegetative reproduction, and is probably accompanied by inter-cytotype mating, which results in almost continuous variation in chromosome numbers and genome size at and above the tetraploid level. Indeed, pollination and hybridization experiments by Lövkvist (1956) revealed weak reproduction barriers between the polyploids within the *C. pratensis* complex. Still, we observed that mixed-ploidy populations were relatively rare (constituting about 10% of populations). Based on their distribution patterns we assume that they have arisen both by secondary contacts between the two cytotypes (in the case of diploids and tetraploids) and by multiple *in situ* polyploidization events (in the case of tetraploids and hexaploids). Populations with the co-occurrence of diploids and tetraploids were found only in the western part of the study area, where these ploidy levels grow largely intermingled, enabling and furthering their secondary contacts. By contrast, tetraploid-hexaploid populations were found scattered in areas occupied by tetraploid populations, suggesting that the hexaploids originated multiple times within those populations. A similar scenario of recurrent hexaploid formation within tetraploid populations has been inferred in ferns based on their C*x* value variation (Hanušová et al., 2019).

In congruence with the phylogenetically distinct position of *C. rivularis* (Franzke and Hurka, 2000), this species had significantly larger monoploid genome size than the other species analyzed here, namely *C. matthioli*, *C. majovskyi*, and *C. pratensis*, which, in turn, possessed highly similar monoploid genome sizes. Still, we found large genome size variation within *C. rivularis*, which apparently reflects various evolutionary processes. First, some genome size divergence was observed between the Romanian and Bulgarian range of this (sub)alpine species, which was probably facilitated by their long-term disjunction (postglacial at least) and evolution in allopatry. As is known from other studies, differential evolution of repetitive DNA and/or selection can generate genome size differences across populations and species, which can be fixed in the absence of gene flow (Bennetzen et al., 2005). As the next cause of the observed genome size variation, triploids have been revealed in the Romanian range (Făgăraş Mts), in agreement with earlier reports (Marhold, 1994a). The formation of unreduced gametes in natural populations is a significant pathway by which triploid cytotypes originate (Mason and Pires, 2015), and it is proposed here also for the case of *C. rivularis*. This species is capable of vegetative propagation (Marhold, 1994a; Ančev, 2006), and, indeed, genetic clones were inferred from the lack of microsatellite variation in both populations from the Făgăraş Mts analyzed here. Vegetative reproduction may therefore explain the successful establishment of triploids and their long-term persistence despite the cytotype minority exclusion phenomenon and sterility issues (Levin, 1975; Ramsey and Schemske, 1998; Husband, 2004; Köhler et al., 2010), similarly as was observed in the triploid *Cardamine × insueta* in the Alps (Zozomová-Lihová et al., 2014b). Much more complex genome size patterns are seen in the Bulgarian range, where apparently both unreduced gamete formation and hybridization with *C. matthioli* have resulted in a mixture of different auto- and allotriploids persisting along with diploids. The substantial genome size variation revealed here within as well as among populations may be due to multiple hybrid formation and even crossing between triploids of different origins, as they seem to be partially fertile (Ančev et al., 2013).

### Multiple Polyploid Origins and Cases of Interspecific Hybridization

Previous authors suggested an autopolyploid origin of *C. majovskyi* based on its occurrence within the area of the diploid *C. matthioli*, morphological as well as molecular data (Marhold, 1994a, 1996; Franzke and Hurka, 2000; Lihová et al., 2003). The genetic patterns resolved here clearly support this scenario, and, in addition, show that the tetraploid *C. majovskyi* arose at least twice. We are thus adding to an ample body of evidence that multiple origins of polyploids are rather the rule than the exception (e.g., Doyle et al., 1990; Soltis et al., 2004; Servick et al., 2015; Rešetnik et al., 2016; Novikova et al., 2017, 2018). Sympatric occurrence of diploids and tetraploids at the same site was rare (Supplementary Data 1). Thus, even if autopolyploids may be recurrently formed within diploid populations, a frequency-dependent mating disadvantage (the principle of minority cytotype exclusion; Levin, 1975) probably hampers common and long-term cytotype co-existence (Kolář et al., 2017; see e.g., Castro et al., 2019), unless they reach a different site or habitat. Even though *C. majovskyi* has not expanded beyond the range of its diploid progenitor so far, it appears to have a significantly different environmental niche. Still, the magnitude of niche divergence is greater between the two genetically and geographically defined lineages spanning both species (i.e., Central and Widespread) than between the two species. Previous studies on other species groups have indicated that autopolyploids may exhibit lower rates of niche evolution than allopolyploids and may consequently persist in geographically close areas and occupy similar niches as their ancestors (e.g., Arrigo et al., 2016; Castro et al., 2019).

Multiple evolutionary scenarios need to be considered to resolve the origins of polyploids of *C. pratensis*. Both diploids and polyploids of *C. pratensis* occur throughout the distribution range of this species (Kučera et al., 2005), as well as within our focus area of Central Europe. Central European diploids grow in several disjunct areas, they are ecologically and genetically diverse, and this heterogeneity provides great potential for multiple polyploidization events. The nested chromosome fusion detected in the hypotetraploid cytotype (2*n* = 30; Mandáková et al., 2013) represents an evolutionarily unique event, which strongly suggests a single origin of hypotetraploids and their subsequent range expansion. The occurrence of this cytotype in all genetic lineages of *C. pratensis*, supposedly descendants from different glacial refugia (see below), also indicates that the polyploidization event predated at least the last glacial period. Unlike *C. majovskyi*, these hypotetraploids have spread widely within as well as beyond Central Europe (Lövkvist, 1956; Lihová et al., 2003, 2004) and typically occupy lowland to montane sites. Hypohexaploids with 2*n* = 44 belonging to the Red lineage, which likely originated from these hypotetraploids, form a stabilized dysploid, genetically and ecologically defined lineage putatively of a single origin (see also Marhold, 1994a,b), although this cytotype has been recorded also in other parts of Europe (Kučera et al., 2005). On the contrary, we can expect multiple origins of other cytotypes at the tetraploid and hexaploid level. Multiple polyploid origins are for instance, supported by a few cases of tetraploid-hexaploid mixed populations, in which the hexaploids apparently originated only recently and *in situ* (discussed above). Tetraploids with 2*n* = 32 were recorded in both the Yellow and Orange lineages, and it is reasonable to assume that they have originated by polyploidization independently in each of them. In contrast to populations with 2*n* = 30, those with 2*n* = 32 grow at higher (sub)alpine sites in the Alps and were recorded also in northern Spain (Lihová et al., 2003). We therefore suggest that the polyploids of *C. pratensis* may have arisen via both single and multiple origins, and probably in earlier (glacial or interglacial) as well as in later (postglacial) periods, as has been documented also in other polyploid complexes (e.g., Brandrud et al., 2020; Rojas-Andrés et al., 2020). Nevertheless, details regarding polyploid origins, such as their source geographic areas, specific diploid or lower-ploidy ancestral lineages and the time of their origin, remain largely unresolved. Franzke and Hurka (2000) proposed that polyploids of the phylogenetic lineage delimited in Central and Northern Europe evolved postglacially. Here we infer that its postglacial origin is highly improbable and that it probably originated in earlier times of Pleistocene glacial-interglacial periods. In fact, the recently published divergence time estimates in the Cardamineae tribe suggests that the entire *C. pratensis* species complex originated at the turn of the Pliocene-Pleistocene (Huang et al., 2020).

The present results have also revealed that, under favorable conditions, the species studied may hybridize. *Cardamine pratensis* is common and widespread in Central Europe, and locally in lowland to montane areas it may grow in close proximity to *C. majovskyi*. A relatively high proportion of genetically admixed populations in their contact zone (found mainly in Slovenia and eastern Slovakia) indicates that gene flow occurs between them. In some of these populations, hybridization was manifested also by increased genome size variation.

Another case of interspecific gene flow was traced between *C. matthioli* and *C. rivularis* in Bulgarian mountains. Even though *C. matthioli* occurs predominantly at lower elevations and only rarely reaches the upper montane belt, and *C. rivularis* is generally a (sub)alpine species (Marhold, 1994b; Ančev et al., 2013), the two species meet in the West Rhodope Mts in Bulgaria. Their co-occurrence and hybridization were recorded at multiple localities within a relatively small area (Ančev et al., 2013), which is confirmed by the present results on three sites in the West Rhodope Mts, and in addition, also in the Vitosha Mts. Ančev et al. (2013) reported morphological intermediacy and triploid chromosome numbers, as well as disrupted meiosis and unbalanced embryological processes in the hybrids. They even suggested apomictic embryo development, but further investigations are needed. It has been assumed that the species contacts and hybridizations occurred relatively recently, as a result of mountain flora disturbance by human activities and because of climatic oscillations (Ančev et al., 2013). Another case of very recent interspecific hybridization in *Cardamine*, triggered by land use changes, has been inferred in the Western Alps (Urbanska et al., 1997; Mandáková et al., 2013; Zozomová-Lihová et al., 2014a).

### Inference of Phylogeographic History

During glacial periods in the Pleistocene, most currently occupied areas in Central Europe were climatically unfavorable for the large-scale survival of temperate species, especially those adapted to moist lowland and montane sites (Hewitt, 2001; Birks and Willis, 2008; Janská et al., 2017). The genetic patterns presented here were presumably shaped by allopatric differentiation when the species ranges became fragmented and their populations survived in more restricted glacial refugia (Hewitt, 2004; Birks and Willis, 2008).

The large genetic variation found in *C. matthioli* and its differentiation into two lineages, strongly suggests that these plants survived the last glaciation in at least two separate refugia. The Central lineage, distributed in a relatively small region spanning from the southeastern margin of the Eastern Alps to northern Croatia, overlaps with an area commonly recognized as a potential glacial refugium and a source area for recolonization by several temperate trees and herbs (e.g., Heuertz et al., 2004, Heuertz et al., 2006; Magri et al., 2006; Bardy et al., 2010; Slovák et al., 2012; Rešetnik et al., 2016; Šrámková-Fuxová et al., 2017; Skokanová et al., 2019). The great diversity and rarity observed currently in populations belonging to the Central lineage support the scenario of *in situ* glacial survival without major population displacement. In contrast to the studies listed above, however, populations of Central lineage did not spread significantly in postglacial times, which resembles the case of *Silene hayekiana* (Durović et al., 2017). The distribution of many species is evidently limited by slow postglacial spreading rather than by environmental limitations (e.g., Svenning and Skov, 2007; Willner et al., 2009; Baer and Maron, 2019). The restricted distribution of the Central lineage stands in strong contrast to the widely extended Widespread lineage. The present-day genetic patterns do not suggest any straightforward hypothesis concerning the glacial survival and postglacial spreading of the latter lineage. Populations in glacial refugia commonly have unique haplotypes and their level of genetic diversity is high because of diversity accumulation and random allele fixation during their long-term persistence (Hewitt, 2000). Large diversity along with high to moderate rarity values in the Western Carpathians suggest that some populations may have survived in microclimatically favorable sites in the foothills of this mountain chain, in so-called ‘cryptic’ or ‘northern’ refugia (Stewart et al., 2010). The Carpathians were only scarcely glaciated (Ronikier, 2011) and hosted fragmented forest communities throughout the last glacial maximum (Jankovská and Pokorný, 2008). The existence of such ‘northern’ refugia has been documented for several temperate species (e.g., Těšitel et al., 2009; Schmickl et al., 2012; Slovák et al., 2012; Kolář et al., 2016; Stachurska-Swakoń et al., 2020). The locations of other potential refugial areas for *C. matthioli* remain unclear, but they may have been patchily scattered across the present range.

The occurrence of *C. majovskyi* in two disjunct areas overlaps with the glacial refugia proposed for its parental diploid lineages, so it seems very likely that *C. majovskyi* originated independently in the same areas where it is currently distributed and that it did not spread significantly afterward. An open question remains if this tetraploid originated during the glacial period or if it has arisen postglacially, which may be supported by its ecological optimum shifted toward higher precipitation and temperatures.

The three longitudinally correlated and ecologically differentiated genetic lineages detected within *C. pratensis* indicate the existence of three separate colonization routes from different refugia, but at the same time substantial genetic admixture among them is suggesting also major postglacial shuffling. In addition, each of them consists of at least two ploidy levels, multiple cytotypes of different origins (e.g., tetraploids with 30 and 32 chromosomes) as well as lowland to subalpine populations, which complicates the reconstruction of their phylogeographic history. Genetic diversity patterns do not indicate any clear geographic trends that are expected for simple scenarios of out-of-refugia colonization routes (Hewitt, 1999; Hewitt, 2000; Nieto Feliner, 2014), but are largely governed by cytotypic distribution patterns. Only the geographically restricted Red lineage (Western Carpathians) appears genetically highly diverse and some populations also exhibited high rarity values, which supports their glacial survival *in situ*, in cryptic microrefugia in the Western Carpathians, similarly as assumed and discussed for *C. matthioli*. Both the more widely distributed Orange and Yellow lineages tend to possess greater rarity values in the Alps and the Carpathians, suggesting that some refugial populations may have survived in their foothills providing favorable humid habitats, followed by northward colonization only postglacially.

Finally, populations of the (sub)alpine taxon *C. rivularis* from the Southern Carpathians and Bulgarian mountains have probably responded to glaciations only by small-scale and elevational range shifts. Mountainous regions in Southern Europe have offered multiple favorable sites for long-term plant survival during climatic oscillations, and the extinction of genotypes and populations was minimized here (Gómez and Lunt, 2007; Nieto Feliner, 2011, 2014). In addition, elevational range shifts may have caused past contacts between *C. rivularis* with *C. matthioli*. Bulgarian populations of *C. matthioli* are genetically slightly differentiated from the rest of the species and exhibit greater affinity to *C. rivularis*. This indicates that contacts and gene flow between *C. matthioli* and *C. rivularis* probably do not occur only at present, but may have happened already in much earlier times.

### Taxonomic Implications

The highly polymorphic species *C. pratensis* has been extensively investigated by many authors since the 1950s, who attempted to split it into more homogeneous entities (e.g., Lövkvist, 1956; Dersch, 1969; Urbanska-Worytkiewicz and Landolt, 1974; Landolt, 1984; Marhold, 1994b; reviewed by Marhold et al., 2018). The genetic patterns revealed in the present study, however, stand in strong contrast with previous taxonomic concepts, as they support none of the putative taxa or entities such as *C. nemorosa*, *C. udicola, C. rivularis* auct. or the ‘ucranica’ morphotype. For instance, morphologically similar (sub-)alpine populations commonly attributed to *C. rivularis* auct. are not genetically closely related and have probably originated multiple times from adjacent lower-elevation populations. Similar scenarios of independent colonization of alpine habitats by several distinct genetic lineages from foothill areas were recently inferred also in *Arabidopsis arenosa* (Kolář et al., 2017) and in the formerly broadly conceived species *Alyssum cuneifolium* (Španiel et al., 2019). In both these cases, the overall phenotypic similarity of alpine populations contrasting with their genetic heterogeneity has been caused by a similar (alpine) environment (Kolář et al., 2017; Monnahan et al., 2019; Španiel et al., 2019; Wos et al., 2019).

The observed genetic structure in *C. pratensis* indicates spatial segregation in the longitudinal direction, but it is not strongly pronounced, with more than one quarter of populations considered inter-lineage hybrids. Genetic differentiation is likely maintained by spatially restricted gene flow, as geographically neighboring lineages are genetically more similar than spatially separated ones. We therefore conclude that there are no strict borders among the lineages, which could potentially serve as basis for a new taxonomic concept, and that the splitting of *C. pratensis*, despite its high genetic, cytotypic and ecological heterogeneity, into segregate taxa cannot be supported.

Although *C. matthioli* and *C. majovskyi* are genetically closely related, which reflects the autotetraploid origin of the latter, their morphological differentiation (Marhold and Záborský, 1986; Lihová and Marhold, 2003), the ecological niche divergence documented here, and rare records of species co-occurrence suggest that the tetraploid represents an independent evolutionary unit, supporting its separate taxonomic treatment (Soltis et al., 2007).

In conclusion, the present study provides detailed insights into the complicated cytotypic and genetic structure of the *C. pratensis* species complex in Central Europe. We identified an interplay of diverse processes that have driven the evolution of the species under study, including allopatric and ecological divergence, hybridization, and multiple polyploid origins in different times, as well as genetic reshuffling caused by Pleistocene climate-induced range dynamics. It is fascinating to see to what extent different evolutionary patterns – regarding cytotypic variation and polyploid evolution as well as phylogeographic scenarios – have evolved in these closely related species.

## Data Availability Statement

The datasets generated for this study can be found in online repositories. The names of the repository/repositories and accession number(s) can be found below: https://data.mendeley.com/datasets/z8tstt9prd/1 and https://www.ncbi.nlm.nih.gov/, PRJNA638616.

## Author Contributions

KM and JZ-L conceived and designed the study. BŠ, KS, TK, MŠ, and KM sampled plant material. AM, TK, MŠ, BŠ, KS, MS, GŠ, JZ-L, MC, TM, ML, and LM generated data and performed data analyses. MŠ, JZ-L, KM, and MS wrote the manuscript. MK designed the Hyb-Seq probes. All authors have read, revised, and approved the final manuscript.

## Conflict of Interest

The authors declare that the research was conducted in the absence of any commercial or financial relationships that could be construed as a potential conflict of interest.

## Footnotes

1 https://github.com/V-Z/sondovac

2 http://tree.bio.ed.ac.uk/software/figtree

3 https://jgi.doe.gov/data-and-tools/bbtools

4 https://github.com/MarekSlenker/snipStrup
