# Supplementary material for: So Closely Related and Yet So Different: Strong Contrasts Between the Evolutionary Histories of Species of the Cardamine pratensis Polyploid Complex in Central Europe
Source: Front Plant Sci. 2020 Dec 18;11:588856. doi: 10.3389/fpls.2020.588856 (PMC7775393; doi:10.3389/fpls.2020.588856)
Supplement: Supplementary file 3 [file Table_1.pdf]

Supplementary Table 1. Characteristics of the microsatellite markers used in the present study. In the loci marked by asterisks, the recorded allele sizes deviated from the expected repeat motifs, and these were scored as 1bp-repeats.

Marker	Repeat motif	Primer sequence (5'→3')		Allele size range (bp)	Total number of	
		forward	reverse		alleles	phenotypes
Cama33	$(AAG)_{8}$	CCGAACCTGAGGAAGTAGGG	GCAGACCTGCATAGCTTGAAC	120-180	10	52
Criv50*	$(AAC)_{7}$	GTCGTATCAAACTCCGCC	ACACAAGAAACCGCAAGATCC	230-310	34	402
Criv12	$(ATC)_{9}$	CAACAGATCTCCCATAGGTCAG	AATCTCAACGGGACACTGG	160-220	10	36
Criv44	$(ACG)_{8}$	GAGAATTGTGAGCTGACGCC	TGGTGGAGGAACTGACCAAC	280-340	14	103
Card19*	$(GAT)_{16}$	AAGGAGTTCCCATCGCCTG	AGATGCTTATATTCCGATGGCG	250-430	58	528
Criv43	$(GTT)_{7}$	GTGCTGCCATGATTGGAC	CAGCTCGAGAAGGCCAAAG	190-260	13	86
Card15*	$(GTT)_{13}$	ACAACCGAATACGTTGGCG	TGGTACCGGAAGAAACTGG	120-190	16	204
Card29*	$(CTTT)_{13}$	GCGACGGAGTTCCAAAGAC	GATCATCTTCCCGCCTCAAG	230-360	31	334
Card4*	$(CTT)_{18}$	CGCAGCTCTCAAGTAAGTG	TCATACCAACGTCAAACGGAAG	100-260	32	371
Criv25*	$(AAG)_{7}$	GGAATATGGTGATCCGAGCC	CTCGCCGTTGCACGAAC	350-450	19	166
Card12	$(AT)_{12}$	ACTCGGAGGAGCAATGGTG	CTTGATCCGCTTGAGAGGC	120-250	5	8
Criv37	$(GAT)_{11}$	AACAAACTGGAGGGAATTACTG	GCAGCCACTCCTAAAGACG	160-220	13	61
Criv5	$(ATC)_{7}$	CTTGCCACTGGAGCACTTG	CAAATCTCGTCTCAGTGTTGG	320-400	17	110
Cama3	$(AAT)_{7}$	CAGAACACCTTCGAGATGGC	TGTGCTTACGCTATTACAAGTCG	190-330	15	111
Card7	$(CTT)_{12}$	ACCTCTTTCGAATCCGGTG	AGCAATCGAGGCTTGTCGG	220-280	13	143
Criv8	$(AAG)_{8}$	GCCCAACCCATTAACTTCGG	TCGTGAGTTACCGGAGAAGC	240-320	19	166
Card17	$(CTT)_{15}$	AGCTTTGCATCCAGCAACC	GGAGAATCAAAGGGAACGGTG	70-310	50	308
Card18	$(AC)_{12}$	CTCACATTGTAACACCAGAAGG	TGCGAGAAGAAGGGTCCAC	270-360	25	313
