# Supplementary material for: So Closely Related and Yet So Different: Strong Contrasts Between the Evolutionary Histories of Species of the Cardamine pratensis Polyploid Complex in Central Europe
Source: Front Plant Sci. 2020 Dec 18;11:588856. doi: 10.3389/fpls.2020.588856 (PMC7775393; doi:10.3389/fpls.2020.588856)
Supplement: Supplementary file 4 [file Table_2.pdf]

Supplementary Table 2. Relative genome size (2C and Cx values are given in arbitrary units, relative to *Solanum lycopersicum* ‘Stupické polní rané’ (2C = 1.96 pg; Doležel et al., 1992)) determined using DAPI flow cytometry for *Cardamine matthioli*, *C. majovskyi*, *C. rivularis* and *C. pratensis*.

	Ploidy level	Number of populations / individuals analysed	Relative genome size (2C) mean (min. – max.)	Monoploid relative genome size (Cx) mean	Variation max. / min. (%)
C. matthioli	2x	28 / 222	0.397 (0.353 - 0.497)	0.198	40.79
C. majovskyi	4x	21 / 205	0.818 (0.736 - 0.903)**	0.204	42.53
C. pratensis	2x	21 / 183	0.421 (0.363 - 0.492)	0.21	35.54
	3x	1 / 1	0.584	0.195	
	4x	61 / 625	0.830 (0.752 - 1.131)	0.208	50.37
	6x	12 / 112	1.263 (1.132 - 1.471)	0.211	29.95
	7x	1 / 7	1.496 (1.387 - 1.536)	0.214	10.74
C. rivularis					
Romania	2x	1 / 13	0.523 (0.518 - 0.543)	0.262	4.738
Bulgaria	2x	3 / 28	0.56 (0.546 - 0.585)	0.28	7.089
Romania	3x	1 / 10	0.793 (0.786 - 0.799)	0.264	1.671
Bulgria	3x*	5 / 37	0.755 (0.681 - 0.865)	0.252	27.061

* Triploids in Bulgaria most likely include both autotriploids of *C. rivularis* and hybrids between *C. rivularis* and *C. matthioli*; see the text for further explanation

** omitting potentially hybrid population Maj_LOG, see the text for further explanation
