# Supplementary material for: So Closely Related and Yet So Different: Strong Contrasts Between the Evolutionary Histories of Species of the Cardamine pratensis Polyploid Complex in Central Europe
Source: Front Plant Sci. 2020 Dec 18;11:588856. doi: 10.3389/fpls.2020.588856 (PMC7775393; doi:10.3389/fpls.2020.588856)
Supplement: Supplementary file 5 [file Table_3.pdf]

Supplementary Table 3. Coefficients of multinomial GLMs with LASSO regularization discriminating among species (model 1), among genetic groups within *Cardamine majovskyi* and *C. matthioli* (model 2) and among genetic lineages within *C. pratensis* (model 3). Only non-zero coefficients are displayed.

	Model 1				Model 2				Model 3		
Variable	*C. majovskyi*	*C. matthioli*	*C. pratensis*	*C. rivularis*	*C. majovskyi* (Central)	*C. matthioli* (Central)	*C. majovskyi* (Widespread)	*C. matthioli* (Widespread)	*C. pratensis* (Red)	*C. pratensis* (Orange)	*C. pratensis* (Yellow)
Topography											
elev				4.02							
exposure	0.03	-0.15			0.02	-0.34				-0.60	0.31
inclination	0.44	-0.02	-0.38	0.02	0.07			-0.40		-0.49	0.58
Temperature											
temp_01			1.04		0.22		-1.71			-0.72	
temp_02											
temp_03											
temp_04	1.98		-2.57								-0.23
temp_05								-1.70			-0.63
temp_06		0.04									
temp_07											
temp_08											
temp_09											
temp_10											
temp_11											
temp_12		-0.31	1.04								
Precipitation											
precip_01		-0.22								-1.22	
precip_02											
precip_03											
precip_04				0.53							
precip_05		0.18						1.04		-0.88	
precip_06	1.16		-1.41		0.38						

	Model 1				Model 2				Model 3		
Variable	*C. majovskyi*	*C. matthioli*	*C. pratensis*	*C. rivularis*	*C. majovskyi* (Central)	*C. matthioli* (Central)	*C. majovskyi* (Widespread)	*C. matthioli* (Widespread)	*C. pratensis* (Red)	*C. pratensis* (Orange)	*C. pratensis* (Yellow)
precip_07			1.43			0.95		-1.27	-0.36		
precip_08			0.13								
precip_09	0.16										
precip_10											
precip_11		0.77	-0.02		1.04						
precip_12	0.44	-0.12									
Solar radiation											
gti_01		-0.60									
gti_02						0.22	-0.67				
gti_03	-0.20	0.81						0.18			
gti_04				-0.24							
gti_05			0.81	-1.01							-1.40
gti_06											
gti_07											
gti_08											
gti_09											
gti_10		-0.35									
gti_11									-2.71		
gti_12				0.64						-0.84	
gti_year											
Photosynthetically active radiation											
par_01	-0.70										
par_02						0.95		-0.40	-1.08		
par_03											
par_04		0.28								-0.26	
par_05									0.83		
par_06								0.39	-0.32		0.57

Variable	Model 1				Model 2				Model 3		
	C. majovskyi	*C. matthioli*	*C. pratensis*	*C. rivularis*	*C. majovskyi* (Central)	*C. matthioli* (Central)	*C. majovskyi* (Widespread)	*C. matthioli* (Widespread)	*C. pratensis* (Red)	*C. pratensis* (Orange)	*C. pratensis* (Yellow)
par_07											
par_08	1.64		-1.82				0.85			-0.62	
par_09	-0.30										
par_10				0.57						0.11	
par_11	0.97		0.00								
par_12	-1.32									-1.12	
par_year											
Bioclimatic variables											
annual Mean Temperature											
mean Diurnal Range		0.26							1.08		
isothermality			-0.07		0.03			-0.07	0.92	-1.23	
temperature Seasonality	0.96					-0.01	0.23				
max Temp WarmestMonth		0.69									
minTemp ColdestMonth			-0.54						-2.43		1.92
tempAnnual Range								0.09			
meanTemp WettestQuarter		0.34				0.26			-1.10	0.87	
meanTemp DriestQuarter		0.85		-0.35					-0.75		
meanTemp WarmestQuarter											
meanTemp ColdestQuarter											
annual Precipitation				-0.61							
precip WettestMonth										1.66	
precip DriestMonth		-0.23									

	Model 1				Model 2				Model 3		
Variable	*C. majovskyi*	*C. matthioli*	*C. pratensis*	*C. rivularis*	*C. majovskyi* (Central)	*C. matthioli* (Central)	*C. majovskyi* (Widespread)	*C. matthioli* (Widespread)	*C. pratensis* (Red)	*C. pratensis* (Orange)	*C. pratensis* (Yellow)
precip Seasonality			0.31				-0.51			0.58	
precip WettestQuarter		0.20									
precip DriestQuarter		-0.36	0.95								
precip WarmestQuarter				-0.36				-0.23		1.51	
precip ColdestQuarter											
