# Supplementary material for: So Closely Related and Yet So Different: Strong Contrasts Between the Evolutionary Histories of Species of the Cardamine pratensis Polyploid Complex in Central Europe
Source: Front Plant Sci. 2020 Dec 18;11:588856. doi: 10.3389/fpls.2020.588856 (PMC7775393; doi:10.3389/fpls.2020.588856)
Supplement: Supplementary file 6 [file Image_1.pdf]

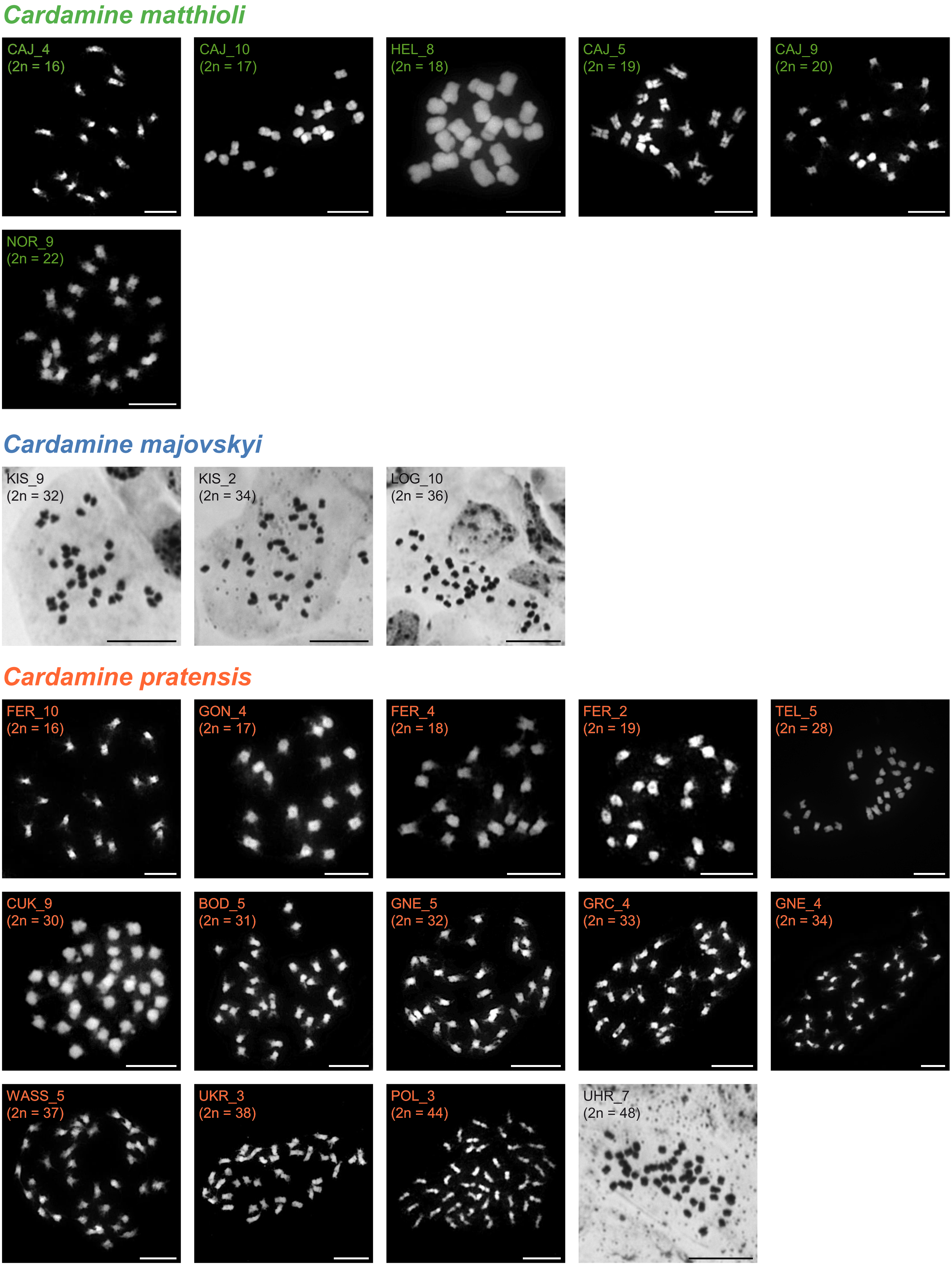

Supplementary Figure 1. Examples of mitotic chromosome spreads stained by DAPI or using the Giemsa stain in selected accessions of the *Cardamine pratensis* complex demonstrating the extensive variation in chromosome numbers. For accession codes see Supplementary Data 1. Scale bars indicate 10 µm.
