# Supplementary material for: So Closely Related and Yet So Different: Strong Contrasts Between the Evolutionary Histories of Species of the Cardamine pratensis Polyploid Complex in Central Europe
Source: Front Plant Sci. 2020 Dec 18;11:588856. doi: 10.3389/fpls.2020.588856 (PMC7775393; doi:10.3389/fpls.2020.588856)
Supplement: Supplementary file 7 [file Image_2.pdf]

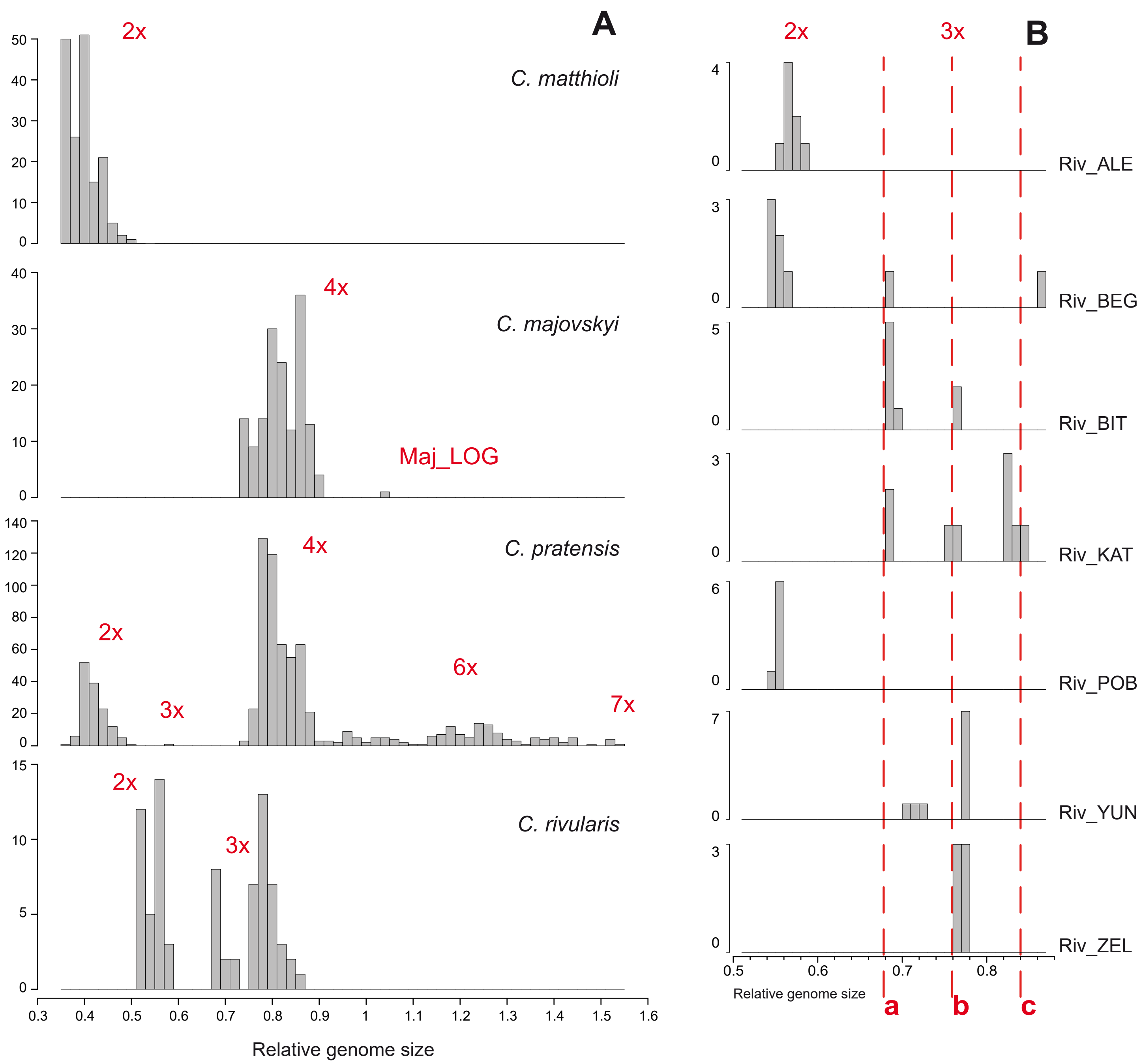

Supplementary Figure 2. Relative nuclear DNA content (2C values) of the *Cardamine* taxa studied (A) and populations of *C. rivularis* from Bulgaria affected by hybridization with *C. matthioli* (B), expressed in arbitrary units (a.u.) relative to the internal standard. Dashed lines in B indicate expected genome sizes of triploids derived from the fusion of: a) a reduced gamete of *C. rivularis* and an unreduced gamete of C. *matthioli* (2C ~ 0.678); b) an unreduced gamete of *C. rivularis* and a reduced gamete of *C. matthioli* (2C ~ 0.759); c) reduced and unreduced gametes of *C. rivularis* (2C ~ 0.84). For locality codes see Supplementary Data 1.
