# Supplementary material for: So Closely Related and Yet So Different: Strong Contrasts Between the Evolutionary Histories of Species of the Cardamine pratensis Polyploid Complex in Central Europe
Source: Front Plant Sci. 2020 Dec 18;11:588856. doi: 10.3389/fpls.2020.588856 (PMC7775393; doi:10.3389/fpls.2020.588856)
Supplement: Supplementary file 8 [file Image_3.pdf]

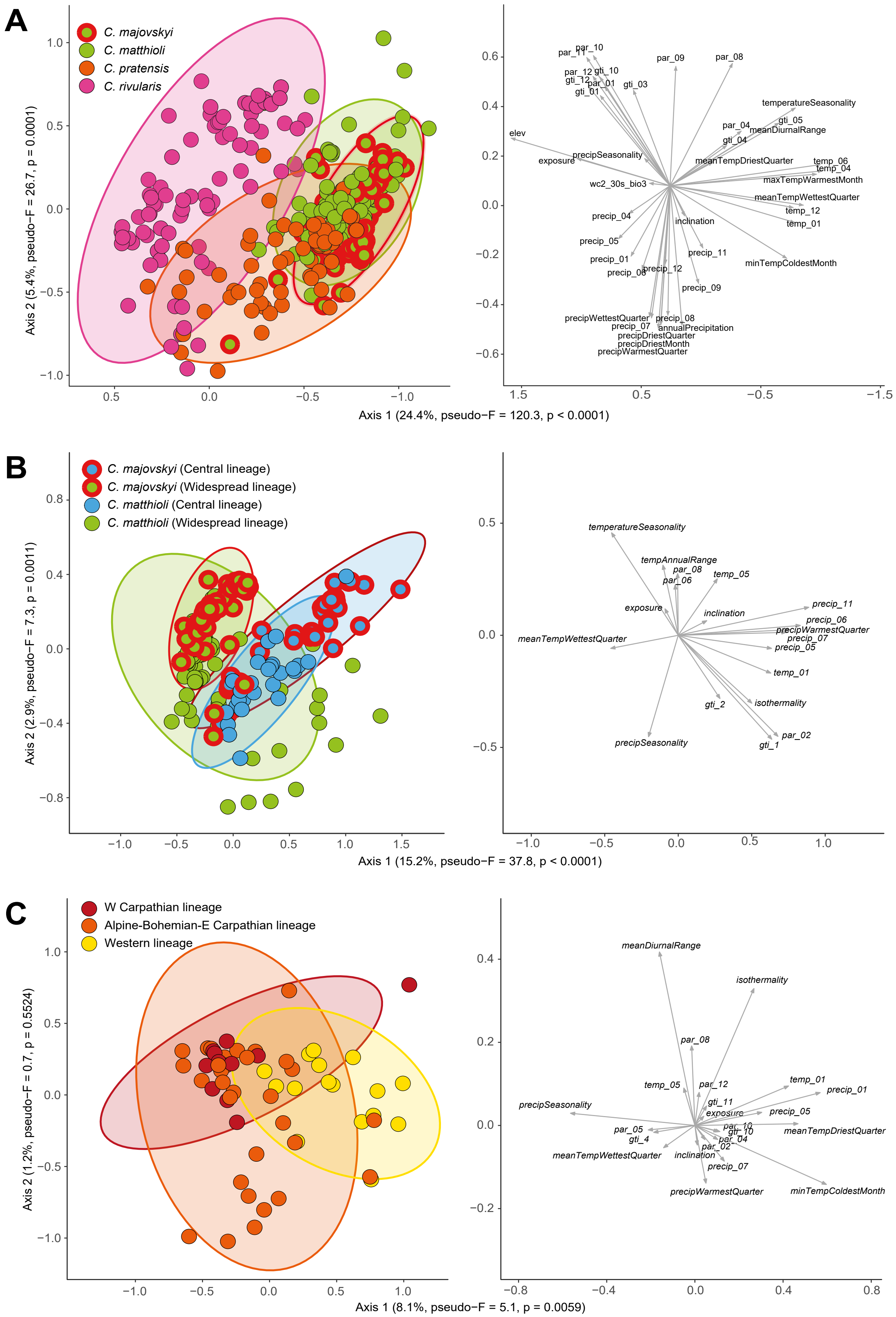

Supplementary Figure 3. Results of RDA showing significant differences in environmental niches of the analysed *Cardamine* species in Central Europe (A), genetic lineages within *C. matthioli* and *C. majovskyi* (B), and three genetic lineages within *C. pratensis* (C). Positions of sampling sites (circles) with species- (or lineage-) specific 95% confidence ellipses (left) and vectors of habitat characteristics (right) are presented. Only characteristics with non-zero coefficients in multinomial LASSO model are shown (for details see Supplementary Table 3). Variance explained by the ordination axes and results of randomization tests are given in parentheses. The scaling of the ordination plots is focused on habitat similarity among sites (left) and on correlations between habitat characteristics (right). For abbreviations of environmental variables see Supplementary Data 2.
